# Stabilization of the RAS:PDE6D Complex Is a Novel Strategy to Inhibit RAS Signaling

**DOI:** 10.1021/acs.jmedchem.1c01265

**Published:** 2022-02-02

**Authors:** Tamas Yelland, Esther Garcia, Charles Parry, Dominika Kowalczyk, Marta Wojnowska, Andrea Gohlke, Matja Zalar, Kenneth Cameron, Gillian Goodwin, Qing Yu, Peng-Cheng Zhu, Yasmin ElMaghloob, Angelo Pugliese, Lewis Archibald, Andrew Jamieson, Yong Xiang Chen, Duncan McArthur, Justin Bower, Shehab Ismail

**Affiliations:** † CRUK Beatson Institute, Glasgow G61 1BD, United Kingdom; ‡ Drug Discovery Program, CRUK Beatson Institute, Glasgow G61 1BD, United Kingdom; § School of Chemistry, University of St Andrews, North Haugh, St Andrews KY16 9ST, United Kingdom; ∥ School of Chemical Engineering and Analytical Sciences, Faculty of Science and Engineering, University of Manchester, Manchester M13 9PL, United Kingdom; ⊥ BioAscent Discovery Ltd, Biocity, Motherwell ML1 5UH, United Kingdom; # Key Laboratory of Bioorganic Phosphorus Chemistry and Chemical Biology, Department of Chemistry, Tsinghua University, Beijing 100084, China; ¶ School of Chemistry, University of Glasgow, Glasgow G12 8QQ, United Kingdom; ∇ Department of Chemistry, KU Leuven, Celestijnenlaan 200G, Heverlee 3001, Belgium

## Abstract

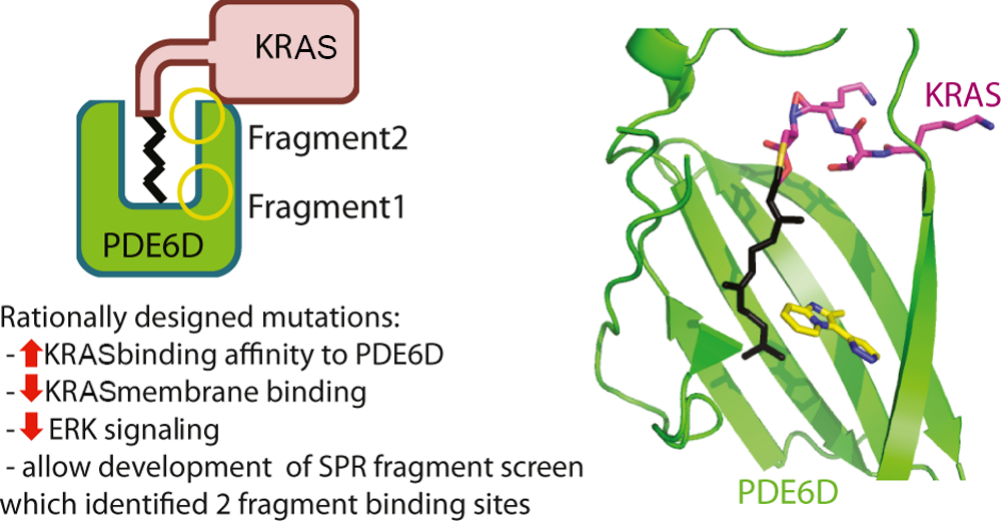

RAS is a major anticancer drug target which requires membrane localization to activate downstream signal transduction. The direct inhibition of RAS has proven to be challenging. Here, we present a novel strategy for targeting RAS by stabilizing its interaction with the prenyl-binding protein PDE6D and disrupting its localization. Using rationally designed RAS point mutations, we were able to stabilize the RAS:PDE6D complex by increasing the affinity of RAS for PDE6D, which resulted in the redirection of RAS to the cytoplasm and the primary cilium and inhibition of oncogenic RAS/ERK signaling. We developed an SPR fragment screening and identified fragments that bind at the KRAS:PDE6D interface, as shown through cocrystal structures. Finally, we show that the stoichiometric ratios of KRAS:PDE6D vary in different cell lines, suggesting that the impact of this strategy might be cell-type-dependent. This study forms the foundation from which a potential anticancer small-molecule RAS:PDE6D complex stabilizer could be developed.

## Introduction

RAS is a family of GTPase proto-oncogenes, comprising four different isoforms: KRAS4A, KRAS4B, HRAS, and NRAS.^1^ All RAS isoforms are ubiquitously expressed, albeit at different quantitative ratios.^2^ These proteins act as molecular switches, whose conformation and hence active state are coupled to their bound nucleotide, either GTP (“on”) or GDP (“off”). RAS has a weak intrinsic GTPase activity and exhibits picomolar binding affinity for nucleotides.^3^ The nucleotide-bound state therefore relies on two types of regulators: guanine nucleotide exchange factors (GEFs) and GTPase-activating proteins (GAPs). Generally, GEFs reduce the binding affinities of small G-proteins to nucleotides, allowing for the fast displacement of the bound nucleotide. GAPs, on the other hand, accelerate the otherwise slow intrinsic GTPase activity of G-proteins, allowing for the fast hydrolysis of GTP to GDP.

Mutations which impair RAS GTPase activity, intrinsic and/or GAP-assisted, promote oncogenesis by shifting its conformation to the GTP-bound “on” state.^4^ The most frequently mutated residues are G12, G13, and Q61.^5^ The importance of RAS in oncogenesis is reflected in the high frequency of cancers containing a RAS mutation. KRAS is most frequently mutated, followed by NRAS and then HRAS.^5^ It is reported that ∼25% of all cancers contain a RAS mutation, with >90% of pancreatic^6^ and a significant percentage of lung and colorectal cancers containing a KRAS mutation. NRAS variants are predominantly found in skin melanoma^7^ and HRAS in head and neck cancer. Consequently, RAS has been a major drug target for over 40 years. Despite these years of efforts to date, only one small molecule (sotorasib) has been granted Food and Drug Administration approval, which specifically targets the RAS G12C mutation.^8−10^ This compound takes advantage of the cysteine mutation to form an irreversible covalent bond with mutant RAS and stabilizes the protein in an inactive state. While this represents a significant advancement in targeting RAS-driven cancers, the G12C mutation is found only in ∼13% of lung adenocarcinoma, 3% of colorectal cancer, and 2% of other solid tumors,^11^ and other oncogenic RAS mutations cannot be treated with this compound. Therefore, new compounds and strategies need to be identified, which can target other oncogenic RAS mutants.

All RAS isoforms contain a C-terminal CAAX motif (cysteine residue followed by two aliphatic residues and any C-terminal residue), a signal that results in the prenylation of the cysteine residue. This post-translational modification is followed by the proteolysis of the three C-terminal (AAX) residues and finally carboxymethylation of the new, C-terminal farnesylated cysteine residue.^12^ The resultant lipid modification increases the affinity of RAS proteins toward membranes, where it is required for signal transduction. Despite the presence of the lipid modification, KRAS has a modest affinity toward membranes, with an average retention time at the plasma membrane of only ∼8 min, whereupon membrane-associated KRAS is “lost” to the cytoplasm through either endocytosis or spontaneous dissociation.^13,14^

Phosphodiesterase 6δ (PDE6D) has a beta-sandwich immunoglobulin fold which contains a hydrophobic pocket capable of binding to the farnesyl group.^15−17^ PDE6D binds to and sequesters the lipid of cytoplasmic RAS and has been proposed to deliver GTPase to recycling endosomes, which is followed by release mediated by the small G-protein Arf-like 2 (ARL2), allowing RAS to be trafficked back to the plasma membrane.^13^ The significance of this trafficking mechanism is reflected in the phenotype of PDE6D knockout cells where KRAS is found localized on endomembranes.^13^ These results generated interest in targeting PDE6D with small-molecule inhibitors. Several chemical scaffolds have been identified that inhibit RAS-PDE6D interactions and thus RAS trafficking, validating PDE6D as a legitimate therapeutic target.^18,19^ Nevertheless, these molecules are yet to make it to the clinic, most likely due to a combination of weak efficiency in inhibiting RAS signaling and off-target effects as they disrupt the trafficking of other prenylated cargoes.^20^ A more nuanced strategy is therefore required to specifically and efficiently target RAS.

Another strategy aimed at disrupting the membrane localization of KRAS blocked farnesylation through the inhibition of farnesyltransferase (FTase).^21,22^ Unfortunately, the inhibition of FTase results in the geranylgeranylation of both KRAS and NRAS and membrane targeting remains unaffected.^23^

**Figure 1:**
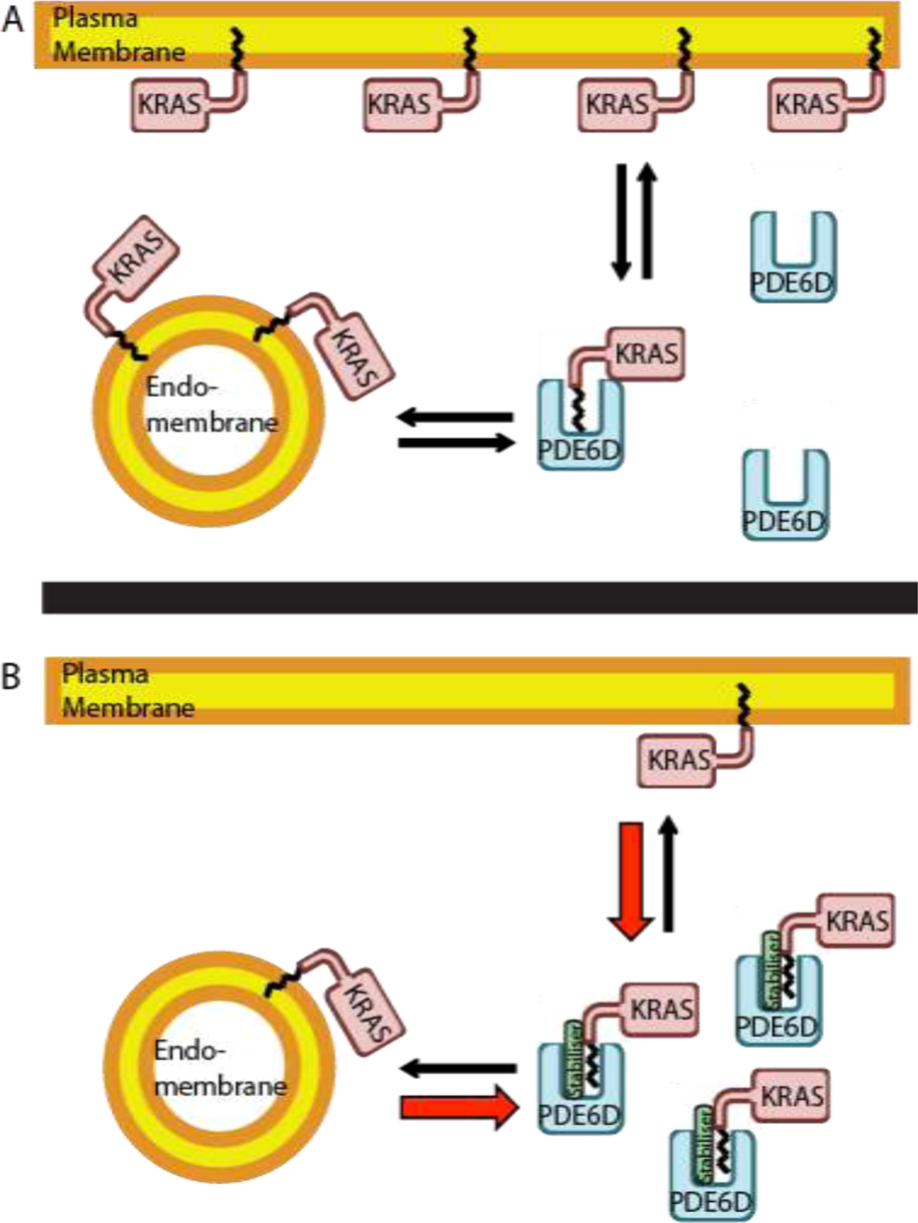
Therapeutic strategy to trap KRAS within the cytoplasm. (A) KRAS is present primarily on the plasma membrane with low concentrations located on endomembranes or bound to PDE6D. PDE6D facilitates KRAS trafficking back to the plasma membrane. Equilibrium constants are represented schematically as black arrows. (B) A small-molecule stabilizer that binds at the PDE6D:KRAS interface shifts the equilibrium constants (red and black arrows) to “trap” PDE6D-bound KRAS in the cytoplasm, away from its binding partners, thus sequestering downstream KRAS signaling.

Recently, a new approach in drug design has emerged with several studies reporting the development of small molecules that stabilize, rather than disrupt, protein–protein interactions.^24−26^ The interaction between the C-terminal tail of RAS and PDE6D presents an opportunity to develop a small molecule which would specifically target the PDE6D:RAS interface to stabilize the interaction, thus shifting the RAS localization equilibrium toward PDE6D and away from the plasma membrane (Figure 1). Indeed, effort has gone into developing small-molecule stabilizers of the PDE6D:KRAS complex, which relied on virtual screening to identify hit compounds; however, there is limited structural or biophysical evidence that these compounds do stabilize the complex.^27,28^

Interestingly, the C-terminal residues of RAS which, in addition to the lipid group, interact with PDE6D as observed in the KRAS:PDE6D cocrystal structure,^29^ are located within the hypervariable region (HVR). The proximity of the PDE6D binding site to a region exhibiting high sequence heterogeneity among the RAS isoforms implies the possibility of fine-tuning the interaction stabilizer to the specific RAS protein.

Here, we show that the stoichiometric ratios of KRAS to PDE6D vary in different cell lines; therefore, such a strategy will most likely have a different impact on different cell types. Furthermore, we present a proof of concept that increasing the affinity of KRAS to PDE6D can relocalize KRAS away from the plasma membrane to disrupt downstream signaling. We were able to extend this concept and developed a fragment-based SPR screening strategy which successfully identified multiple fragments capable of binding to the PDE6D:KRAS complex, as shown through cocrystal structures. This paves the way toward the development of a small-molecule PDE6D:KRAS interaction stabilizer and—potentially—a novel anticancer drug.

## Results

### PDE6D Interacts with Numerous Prenylated Proteins Including KRAS, NRAS, and HRAS

**Figure 2:**
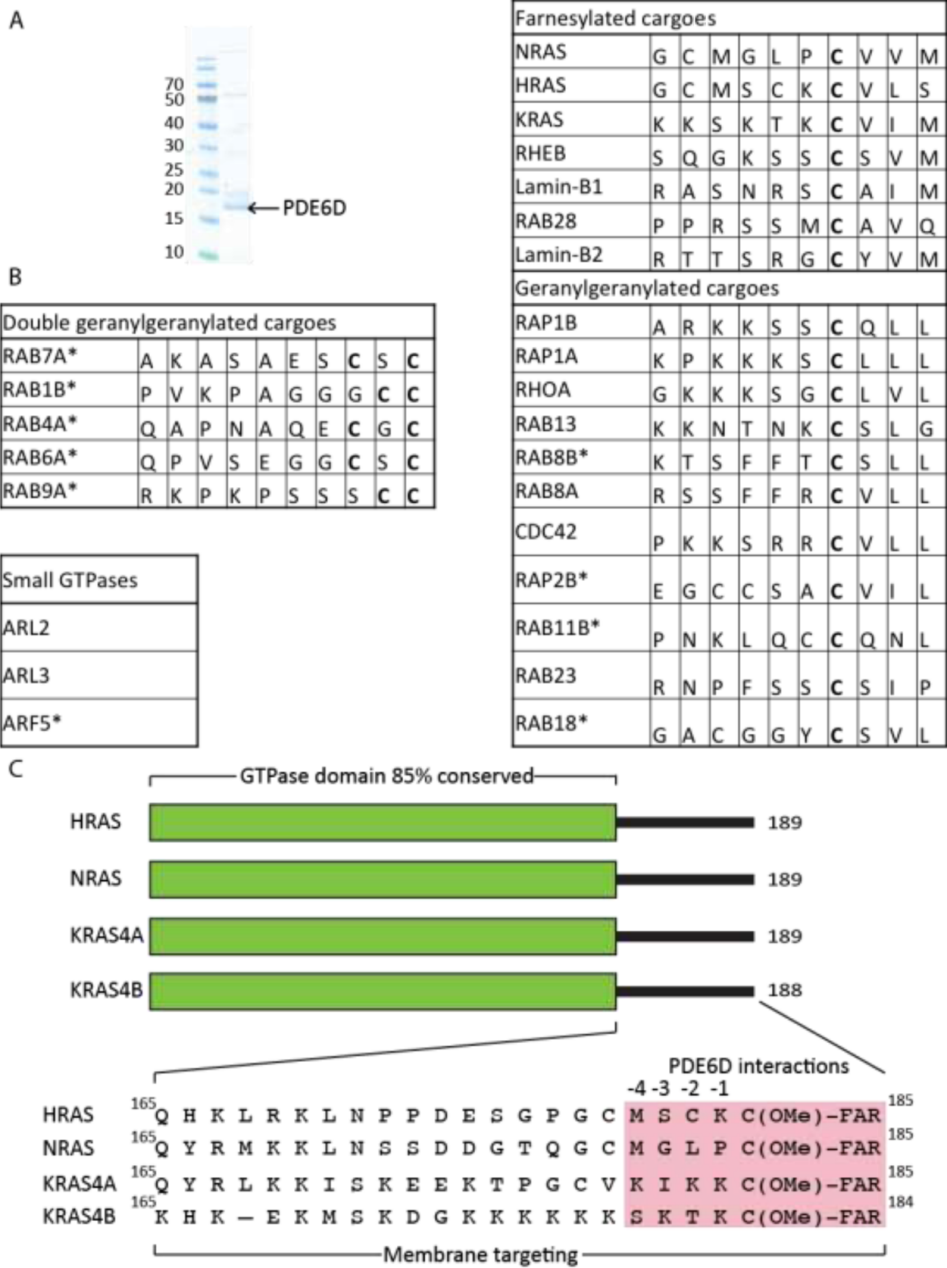
Identification of PDE6D interactors. (A) Sodium dodecyl sulfate–polyacrylamide gel electrophoresis (SDS-PAGE) gel of PDE6D (highlighted with an arrow) with potential interactors coeluted with PDE6D from size-exclusion chromatography. (B) Table of binding partners identified from mass spectrometry analysis; novel interactors are highlighted with an *. The C-terminal sequence of each protein is shown with the post-translationally modified cysteine highlighted in bold. (C) Domain structure of the four RAS isoforms with HVRs aligned. Potential residues involved in PDE6D binding highlighted in pink.

To ensure that PDE6D is capable of binding to and solubilizing multiple RAS isoforms, we used recombinant PDE6D to copurify endogenous proteins from the HEK293F cell lysate (Figure 2). Following proteomic mass spectrometry, KRAS4B (hereafter called KRAS, unless the isoform is specifically mentioned), NRAS, and HRAS were all successfully identified. The absence of KRAS4A may possibly reflect its expression level compared to the other isoforms. Interestingly, in addition to N/H/KRAS, more 4 farnesylated proteins, 10 geranylgeranylated, and 6-double geranylgeranylated binding partners were identified. To determine whether double-geranylgeranylated small G-proteins interact with PDE6D via their GTPase domain or their lipid-modified C-terminus, pulldown experiments using nonprenylated GST-tagged RAB1B were performed; however, no interaction was observed (Figure S1). This suggests that the interaction is most likely mediated by the lipid modification. To our knowledge, this is the first time double-geranylgeranylated proteins have been shown to potentially bind to PDE6D, suggesting that PDE6D may have a broader role in protein trafficking than previously thought, which raises the question whether PDE6D can bind to prenylated, palmitoylated proteins—including NRAS and HRAS.

Finally, three non-prenylated small GTPases were identified: ARL2 and ARL3, which are known to function as PDE6D cargo release factors,^16,30^ and a novel interactor—ARF5. All PDE6D-binding partners identified in this study are listed in Figure 2B.

### Prenylated PDE6D Cargoes Lack a Consensus-Binding Motif

The sequence analysis of all prenylated proteins identified as PDE6D interactors failed to identify a conserved PDE6D-binding motif, with substantial variations in residue charge and size observed within the C-terminal residues that can interact with PDE6D. The only conserved feature is the modified C-terminal cysteine residue. Indeed, the affinities of 13-residue C-terminal peptides of WT KRAS and HRAS for PDE6D (1.3 and 2.7 μM, respectively, Figure S2A,B) are comparable to those reported for full-length KRAS (1–2 μM)^29^ and RheB (∼0.5 μM).^17^ These affinities are also similar to those of a carboxymethylated geranylgeranylated cysteine residue (2.7 μM, Figure S2C) and a carboxymethylated farnesylated cysteine residue (0.9 μM, Figure S2D), suggesting that most residues preceding the lipidated cysteine do not affect binding to PDE6D. A list of the dissociation constants determined in this study is shown in Figure S2E. In contrast, the phosphatase INPP5E, which is trafficked to the primary cilium, binds to PDE6D with a reported affinity of ∼10 nM and it was described as a “high-affinity cargo”.^31^ Characterization of the INPP5E:PDE6D interaction identified two residues that confer high-affinity binding to PDE6D: an isoleucine in the −1 position and a serine in the −3 position relative to the carboxymethylated prenylated C-terminal cysteine.^31^

Both RAS and RheB are released by the release factors ARL2 and ARL3.^13,30^ Interestingly, however, INPP5E is not released by ARL2,^31^ indicating that PDE6D cargo release is linked to the cargo binding affinity. Therefore, while the modified cysteine is sufficient for PDE6D binding, high-affinity cargo-PDE6D interaction is achievable, which could influence protein trafficking and thus be utilized as a tool to alter KRAS localization.

### Rational Design of a KRAS Mutant That Binds to PDE6D with High Affinity and Abolishes Release by ARL2 and ARL3

**Figure 3:**
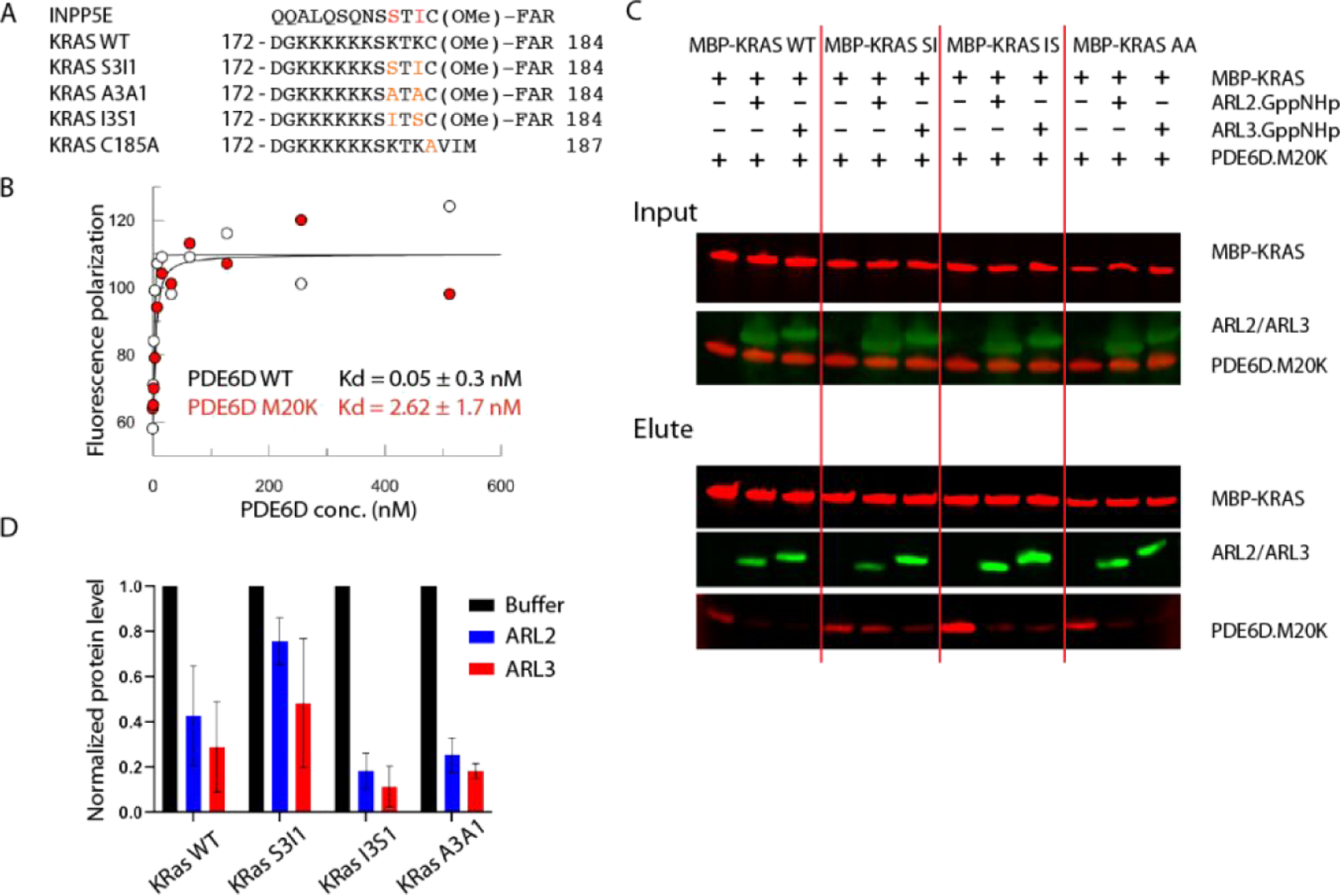
Engineering a KRAS construct with high PDE6D binding affinity. (A) KRAS HVR sequences used in this study and compared to the high affinity PDE6D cargo protein INPP5E. Residues mutated in this study are shown in orange. (B) Fluorescence polarization binding curve for the KRAS S3I1 peptide [fluorescein-DGKKKKKKSSTIC(OMe)-Far] at a concentration of 5 nM with either PDE6D WT (white circles) or PDE6D M20K (red circles) titrated within a concentration range of 0–512 nM with twofold serial dilutions. PDE6D bound the KRAS S3I1 peptide with a dissociation constant of 0.05 ± 0.3 nM, while the *K*_d_ value of the PDE6D M20K mutant was 2.62 ± 1.7 nM. Curves were fitted using Grafit. (C) Representative pulldown and release assay of MBP-KRAS (G12D) WT, MBP-KRASv (G12D) S3I1, MBP-KRAS (G12D) A3A1, and MBP-KRAS (G12D) I3S1 in the absence or presence of ARL2.GppNHp or ARL3.GppNHp. Pulldown quantified in (D). Experiments done in triplicate. KRAS (G12D) WT + ARL2 is 0.42 ± 0.22, KRAS (G12D) WT + ARL3 is 0.29 ± 0.19, KRAS S3I1 + ARL2 is 0.76 ± 0.10, KRAS S3I1 + ARL3 is 0.48 ± 0.28, KRAS I3S1 + ARL2 is 0.18 ± 0.08, KRAS (G12D) WT + ARL3 0.11 ± 0.09, KRAS A3A1 + ARL2 is 0.25 ± 0.08, and KRAS A3A1 + ARL3 0.18 ± 0.03.

To test if the INPP5E high-affinity PDE6D-binding motif can increase the KRAS affinity for PDE6D, we used a fluorescein-labeled 13-residue peptide of a mutant KRAS HVR sequence, which incorporates the Ser-3/Ile-1 motif (Figure 3A). The dissociation constant for the PDE6D interaction with this peptide was determined to be 0.05 ± 0.3 nM (Figure 3B). While such high affinities are challenging to measure accurately, it is clear that the two-point mutations dramatically increase the binding affinity of KRAS to PDE6D, up to 26,000-fold, when compared to WT KRAS.

To compensate for the effect of overexpressing PDE6D in cells, we decided to express an “attenuated” version of PDE6D. The PDE6D M20K mutation reduces the affinity toward cargo proteins but does not abolish it^17^—in agreement with this, the KRAS S3I1 variant binds to PDE6D M20K with an affinity of 2.62 ± 1.7 nM. To test if the increase in the binding affinity inhibits ARL2/ARL3-mediated cargo release with PDE6D M20K, a pulldown and release assay was performed (Figure 3C,D). In this experiment, recombinant MBP-tagged prenylated KRAS(G12D) S3I1 mutant showed less release in the presence of excess ARL2.GppNHp or ARL3.GppNHp compared to MBP-tagged prenylated KRAS(G12D) with a wild-type hypervariable region [hereafter named KRAS(G12D) WT]. To ensure that this effect is specific to the high-affinity mutations, two negative-control constructs were used—a double-alanine mutant [KRAS(G12D) A3A1] and a variant with the affinity-modulating residues in reverse [KRAS(G12D) I3S1]. These KRAS variants bind to WT PDE6D with affinities of 1.6 and 0.7 μM, respectively (Figure S3). Both of these negative-control mutations allow ARL2/ARL3-mediated PDE6D release (Figure 3C,D), which demonstrates that the inhibition of ARL2/ARL3 activity is directly linked to the high affinity binding of the KRAS(G12D) S3I1 variant to PDE6D.

### Crystal Structure of the PDE6D:KRAS S3I1 Complex

**Figure 4:**
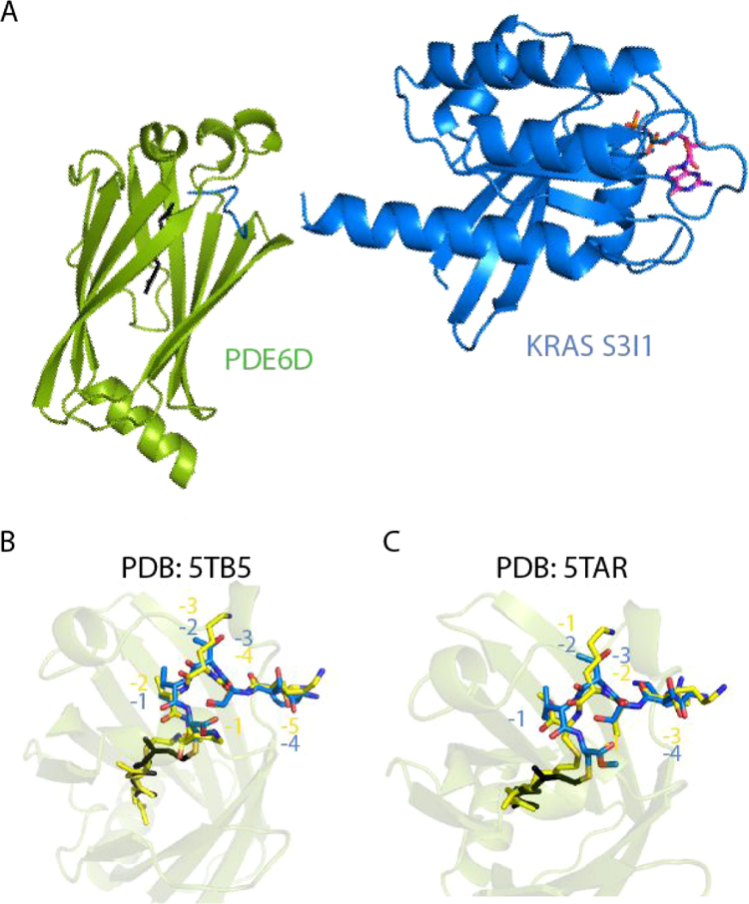
Structural characterization of the PDE6D: KRAS S3I1 interaction. (A) Crystal structure of the WT PDE6D:KRAS S3I1 complex at a 2.2 Å resolution (PDB: 7Q9U). KRAS is shown in a cartoon form in blue with the bound GDP nucleotide in a pink stick form. PDE6D is shown in green. The farnesyl group is represented in a stick form in black and is buried within the PDE6D cargo-binding pocket. (B,C) Comparison of the PDE6D-binding residues of the KRAS S3I1 mutant with the existing PDE6D:KRAS cocrystal structures [PDB: 5TB5, (B) and 5TAR, (C)]. The high-affinity mutant is shown in a stick form in blue, while 5TB5 and 5TAR are shown in yellow. Residues are numbered relative to the modified cysteine residue.

Having confirmed that the KRAS S3I1 mutant binds to PDE6D with high affinity, we sought to further characterize this altered interaction. To this end, we determined the crystal structure of the full-length KRAS S3I1 mutant in complex with PDE6D at a 2.2 Å resolution (Figure 4A).

The crystal structure contains two copies of the PDE6D:KRAS S3I1 mutant complex within the asymmetric unit, where each KRAS molecule binds to PDE6D in the same conformation. Comparing our new structure to the complex of INPP5E C-terminal peptide bound to PDE6D (PDB: 5F2U), we can see that the binding conformation of the PDE6D interacting residues is conserved (Figure S4).

We next sought to compare the binding conformation of our high-affinity complex to the KRAS WT:PDE6D complex (PDB: 5TAR and 5TB5—Figure 4B,C). When the structures are overlaid, we can see that the backbone positions are conserved for residues 180–184 of KRAS with a root-mean-square deviation of 0.42 Å for Cα atoms compared to PDB: 5TAR and 0.44 Å for Cα atoms relative to PDB: 5TB5. With the high level of conservation in the peptide backbone conformation, particularly of the surface-exposed residues, the high-affinity KRAS S3I1 mutant may serve as a surrogate for the KRAS WT:PDE6D complex.

### KRAS(G12) S3I1 Mutation Shifts the Equilibrium toward PDE6D Binding, Precluding Membrane Association and Disrupting Erk Signaling

**Figure 5:**
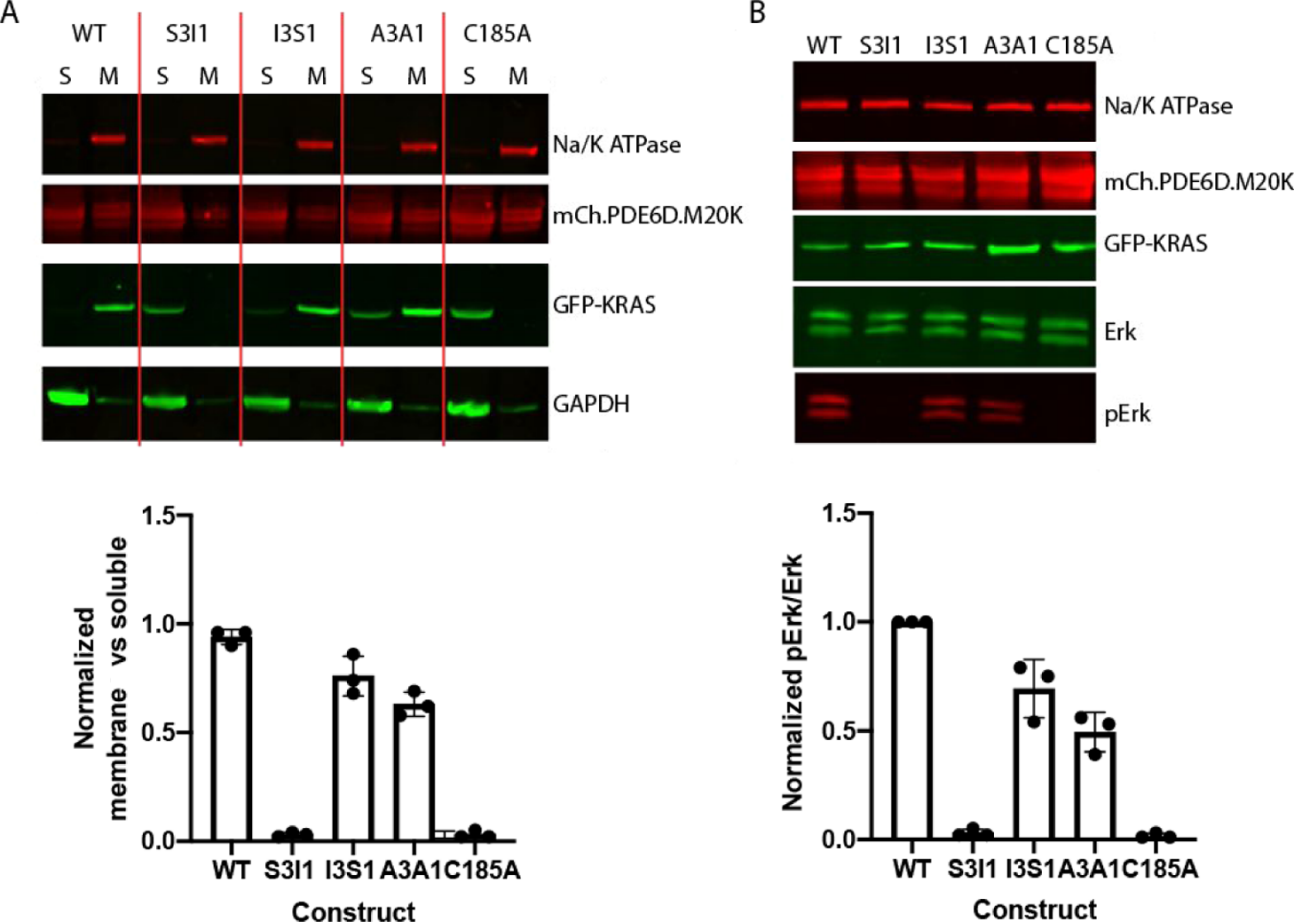
Increasing the affinity to PDE6D prevents KRAS membrane association and reduces phospho-Erk levels. (A) Cellular fractionation of HEK293F cells coexpressing mCherry-PDE6D M20K and KRAS(G12D) constructs (WT, S3I1, I3S1, A3A1, and C185A) showing the ratio of membrane and soluble fractions of each KRAS construct. The immunoblots in (A,B) are representative of three independent experiments. Data are quantified in the bar charts below. (B) Phospho-Erk levels in HEK293F cells cotransfected with mCherry PDE6D M20K and KRAS (G12D) S3I1 are significantly lower relative to G12D WT and negative controls and comparable to the positive KRAS control, C185A.

Having demonstrated the dramatic increase in the binding affinity to PDE6D and concomitant inhibition of ARL2/ARL3 release of the KRAS(G12D) S3I1 construct, we sought to determine the effect of these mutations on KRAS localization and downstream signaling in cells that were double-transfected with oncogenic G12D KRAS (WT, S3I1, S1I3, A3A1, or a CAAX mutant) and PDE6D M20K (Figure 5).

Cell fractionation experiments were performed to compare the levels of the different KRAS constructs in the membrane and soluble fractions. As seen in Figure 5A, by introducing the KRAS mutations that increase the binding affinity to PDE6D, we were able to successfully solubilize KRAS(G12D) S3I1 protein and retain it within the cytoplasm. Both negative-control constructs [KRAS(G12D) I3S1 and A3A1] showed some degree of solubilization relative to the KRAS(G12D) WT; however, this is most likely due to the loss of two lysine residues within the positively charged HVR, which can reduce KRAS affinity for membranes,^32^ rather than due to altered interactions with PDE6D. In fact, in the absence of PDE6D overexpression in the cells (Figure S5), KRAS S3I1 is predominantly associated with the membranes, which demonstrates that cytoplasmic localization is primarily a result of tight binding to PDE6D and not reduced membrane affinity or faulty CAAX box processing. Furthermore, the proportion of soluble KRAS(G12D) A3A1 and I3S1 proteins is low, while the high-affinity S3I1 variant appears to be de facto absent from the membrane fraction analogously to the positive-control construct C185A, which lacks the CAAX box and is therefore not prenylated.

Having demonstrated that increasing the binding affinity to PDE6D can effectively relocalize KRAS away from the plasma membrane, we next sought to determine if the “relocalization” of KRAS altered downstream signaling. Erk is a serine/threonine kinase downstream of KRAS, which becomes phosphorylated upon activation.^33^ When the different KRAS variants harboring the oncogenic mutation G12D were transfected into the cells in the absence of PDE6D, the ability of KRAS to activate downstream signaling via Erk was clearly evident, except in the case of the nonprenylated KRAS(G12D) C185A variant (Figure S5B). Nevertheless cotransfection of PDE6D M20K together with KRAS (G12D) S3I1 resulted in significant reduction of the phospho-Erk levels (Figure 5B). We conclude that trapping KRAS in the cytoplasm is an almost complete abrogation of downstream signaling as reflected in the very low levels of Erk phosphorylation (Figure 5B), which suggests that there is therapeutic potential to stabilize the PDE6D:KRAS interaction.

### High-Affinity KRAS Traffics to the Primary Cilia in the Presence of PDE6D

**Figure 6:**
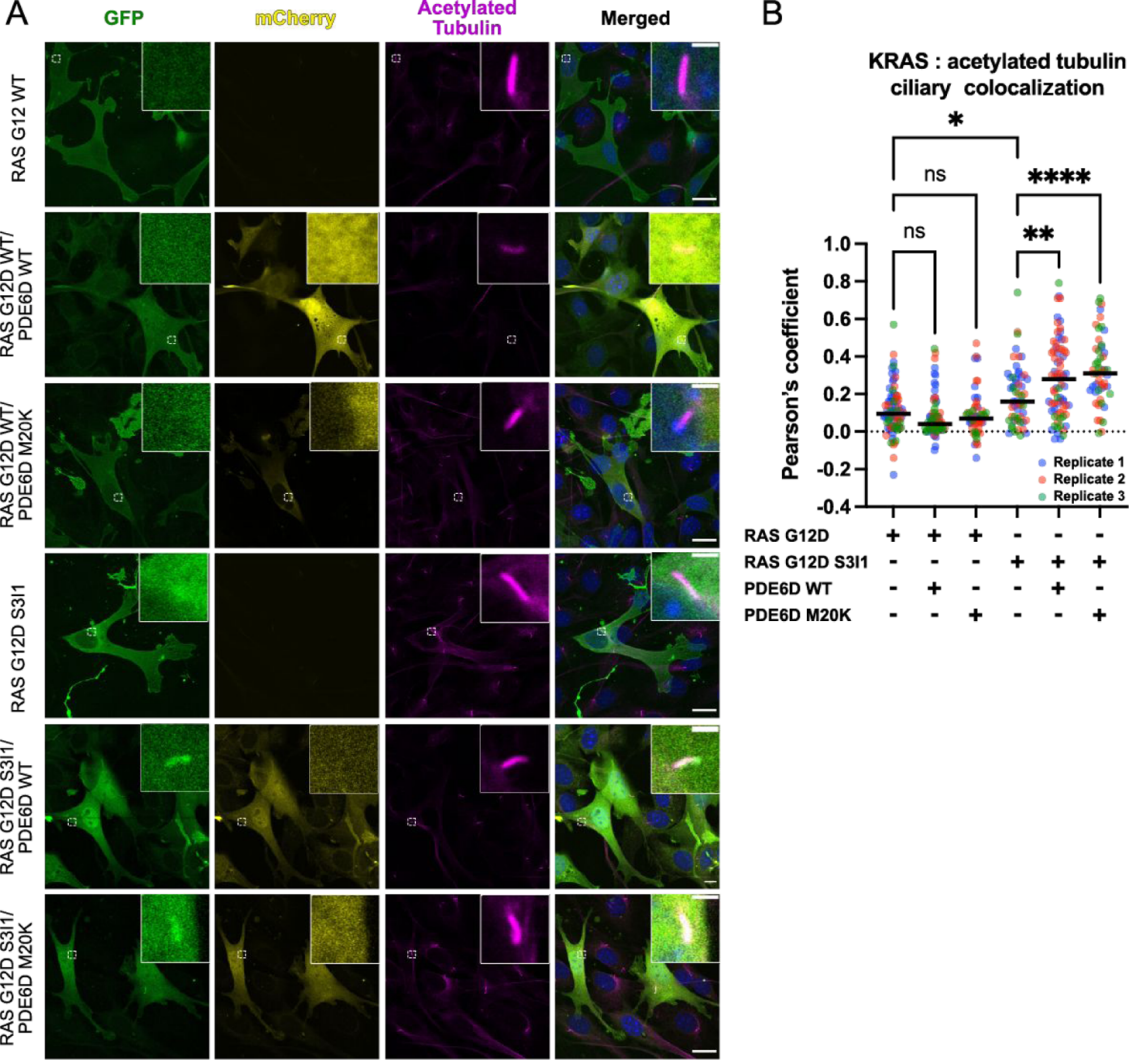
Ciliary localization of high-affinity KRAS (G12D) S3I1 in the presence of PDE6D. (A) Representative confocal images of NIH-3T3 cells expressing KRAS (GFP, in green) alone or together with PDE6D (mCherry, in yellow). Cells were plated on 1.5 glass coverslips 18–24 h after transfection and allowed to attach overnight. After 24 h of starvation with 0.5% fetal calf serum-containing media to induce ciliation, the cells were fixed using 4% paraformaldehyde and immunolabelled for acetylated tubulin (magenta) to identify primary cilia. The images shown are the maximum *z*-projections of three optical sections (1 μm thick) acquired at a 0.5 μm *z*-interval. Scale bars are 20 μm, insets 2 μm. (B) Quantification of ciliary localization of exogenous KRAS in the presence or absence of exogenous PDE6D. The accumulation of KRAS at the primary cilium was estimated by examining the colocalization of KRAS and acetylated tubulin in 5 μm^2^ regions of interest (ROIs) containing cilium using Pearson’s coefficient. For each condition, at least 15 ROIs (15 cells) were analyzed in three independent experiments. All individual values are shown together with the median (black lines). Asterisks relate to the *p*-value: * ≤ 0.05, ** ≤ 0.01, **** ≤ 0.0001 by one-way ANOVA and Šídák’s multiple comparison test.

Since PDE6D plays a critical role in trafficking high-affinity cargoes to the primary cilia,^31^ it is possible that increasing the affinity of KRAS to PDE6D could relocalize KRAS to the primary cilium. To investigate this possibility, we examined the localization of GFP-tagged KRAS(G12D) S3I1 in ciliated cells by confocal microscopy. Using acetylated tubulin as a marker for primary cilium, we examined the colocalization levels of GFP (KRAS) and acetylated tubulin as a measure of KRAS-specific localization to the cilium. Thus, when we overexpressed GFP-tagged KRAS(G12D) WT alone or together with mCherry-tagged PDE6D WT or PDE6D M20K, we observed no particular accumulation of KRAS at the cilia (Figure 6A) and neither colocalization of KRAS and tubulin (low Pearson’s coefficient, Figure 6B). Unexpectedly, KRAS did not show enrichment at the plasma membrane and was predominantly cytosolic, most likely due to the high levels of KRAS overexpression, which disrupted the native localization of KRAS. We however observed some accumulation in the areas surrounding the cilia when overexpressing KRAS(G12D) S3I1 alone, as can be observed by a small but significant increase in Pearson’s coefficient when compared to KRAS(G12D) WT overexpression. Moreover, when KRAS(G12D) S3I1 was coexpressed with either PDE6D WT or PDE6D M20K, we found a clear ciliary localization (Figure 6A) and a significant increase in KRAS and tubulin colocalization by means of a significant increase in Pearson’s coefficient when compared to that of KRAS (G12D) WT or S1I3 alone (Figure 6B). We conclude that high-affinity KRAS traffics to the primary cilia in the presence of PDE6D.

### PDE6D:KRAS Stoichiometric Ratios in Cells

**Figure 7:**
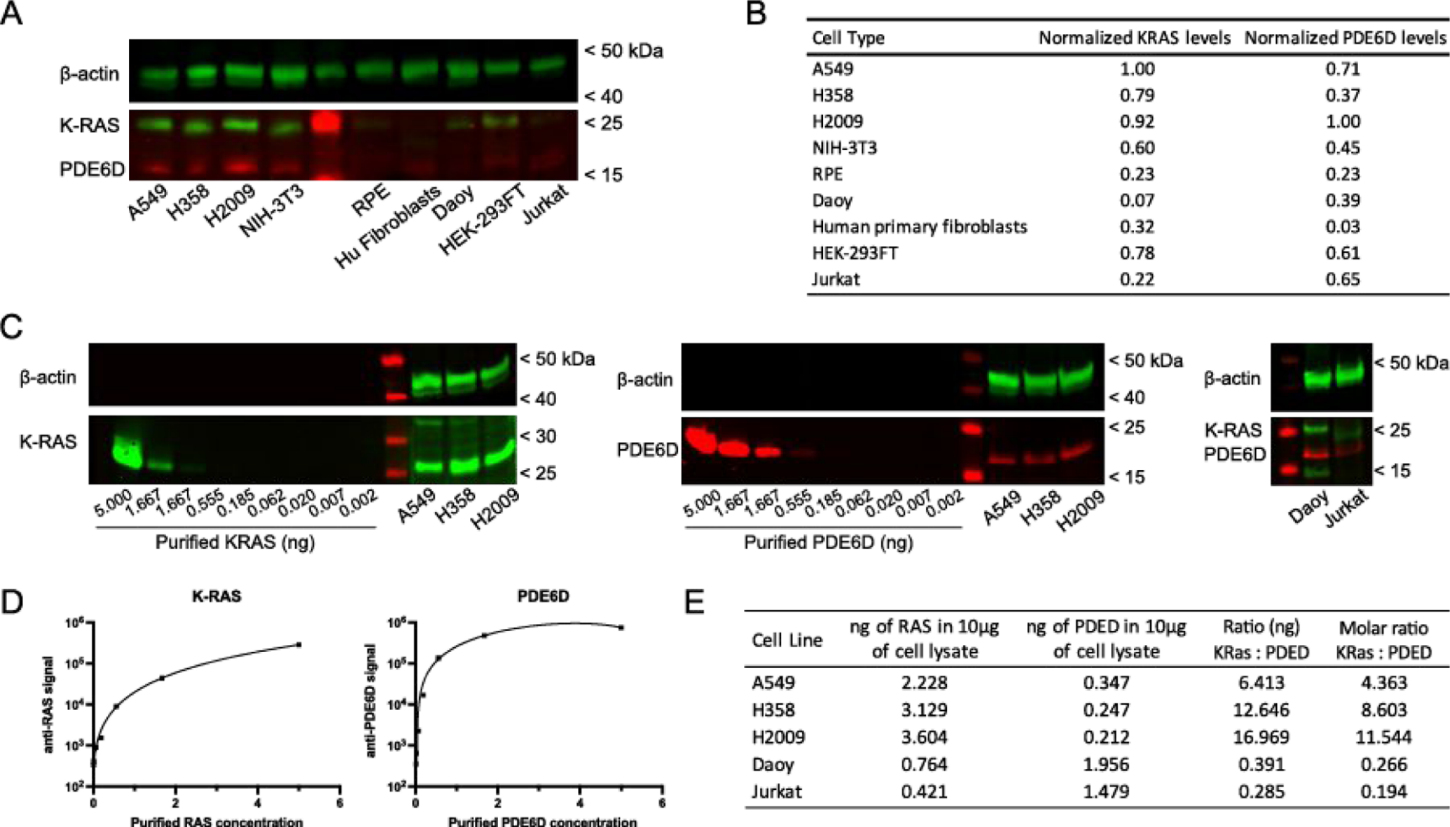
Relative abundance and molar ratio of KRAS and PDE6D in cell lines. (A) Western blot analysis of KRAS and PDE6D endogenous levels in human lung cancer cells (A549, H358, and H2009), murine fibroblasts (NIH-3T3), human retinal cells (RPE), human primary fibroblasts, human cerebellar medulloblastoma cells (Daoy), human kidney cells (HEK-293FT), and human lymphoma cells (Jurkat). Anti-KRAS monoclonal antibody WH00034845M1 (SIGMA) and anti-PDE6D polyclonal antibody CSB-PA526126LA01HU (CUSABIO, Houston, TX, USA) were raised against the human proteins. The same antibodies were used for the murine cell line NIH-3T3, where KRAS and PDE6D showed 97.3 and 98% sequence identity with the human proteins, respectively (B). Normalized levels of KRAS and PDE6D shown in (A). (C) Correlation between the fluorescent signal of anti-KRAS or anti-PDE6D antibodies and the amount of purified KRAS or PDE6D protein examined by western blotting. In the left panel: standard curve using KRAS protein and endogenous KRAS levels of cancer cells A549, H358, and H2009. In the central panel: standard curve using PDE6D protein and endogenous PDE6D levels of A549, H358, and H2009. In the right panel: endogenous levels of KRAS and PDE6D in Daoy and Jurkat cells. Unless otherwise specified, all western blot analyses were done using 10 μg of the cell lysate per lane. (D) Nonlinear fitting of KRAS and PDE6D standard curves from (C) (GraphPad Prism 9). (E) Estimation of endogenous KRAS and PDE6D levels [from (C)], KRAS:PDE6D mass ratio, and KRAS:PDE6D molar ratio by the extrapolation of nonlinear fitting of the standard curve.

Our in vitro assays support that the stabilization of the PDE6D/KRAS complex could be a successful strategy with therapeutic potential; however, an important factor we should not overlook is the influence of the stoichiometric ratio of both molecules and how this could affect the efficiency of the reaction and the off-target effects. If KRAS levels are much higher than PDE6D, this can result in a low-efficiency approach. Furthermore, the stabilization would result in the high occupancy of PDE6D and thus would result in inhibiting PDE6D-mediated trafficking. To tackle this, we analyzed the stoichiometry of KRAS and PDE6D in cells. For that, we examined the relative levels of KRAS and PDE6D in different cell types. We found that KRAS and PDE6D relative levels are widely variable among different cell types, with the highest levels of both proteins in human cancer cells (A549, H358, and H2009) and their lowest levels in retinal retinal pigmented epithelial (RPE) cells (Figure 7A,B).

We examined the ratio of KRAS:PDE6D in three lung cancer cell lines that had the highest KRAS levels among the cells analyzed (A549, H358, and H2009), as well as two cell lines with low KRAS levels but seemingly higher levels of PDE6D than KRAS (Daoy and Jurkat). To do this, we loaded known concentrations of purified KRAS or PDE6D and preselected cell lysates (Figure 7C). Using a nonlinear fitting model (Figure 7D), we extrapolated the fluorescence levels obtained and calculated the amount of KRAS and PDE6D in each cell lysate, as well as the mass ratio and molar ratio of KRAS:PDE6D (Figure 7E). The molar ratio for KRAS:PDE6D ranged from ∼0.2 to 12 times depending on the cell line. This might indicate that this strategy will have a different impact on different cell lines with the most likely benefit in cells with low KRAS:PDE6D ratios. The cells with higher KRAS:PDE6D ratios might not benefit from this strategy with the possibility of adverse effects due to the high occupancy of PDE6D with KRAS. Nevertheless, the relocalization of KRAS to the cilia in ciliated cells might result in the cycling of PDE6D and the presence of free PDE6D even in the presence of high levels of high-affinity-binding KRAS to PDE6D. This is based on the reports that proposed that active ARL3 is rich in the cilia, which would allow the release of KRAS and unoccupied PDE6D.^34,35^ However, this observation warrants deeper investigation as the effect of enriching KRAS in primary cilia is unknown.

### Identification of Small-Molecule Fragments That Bind to the WT KRAS:PDE6D Complex

**Figure 8:**
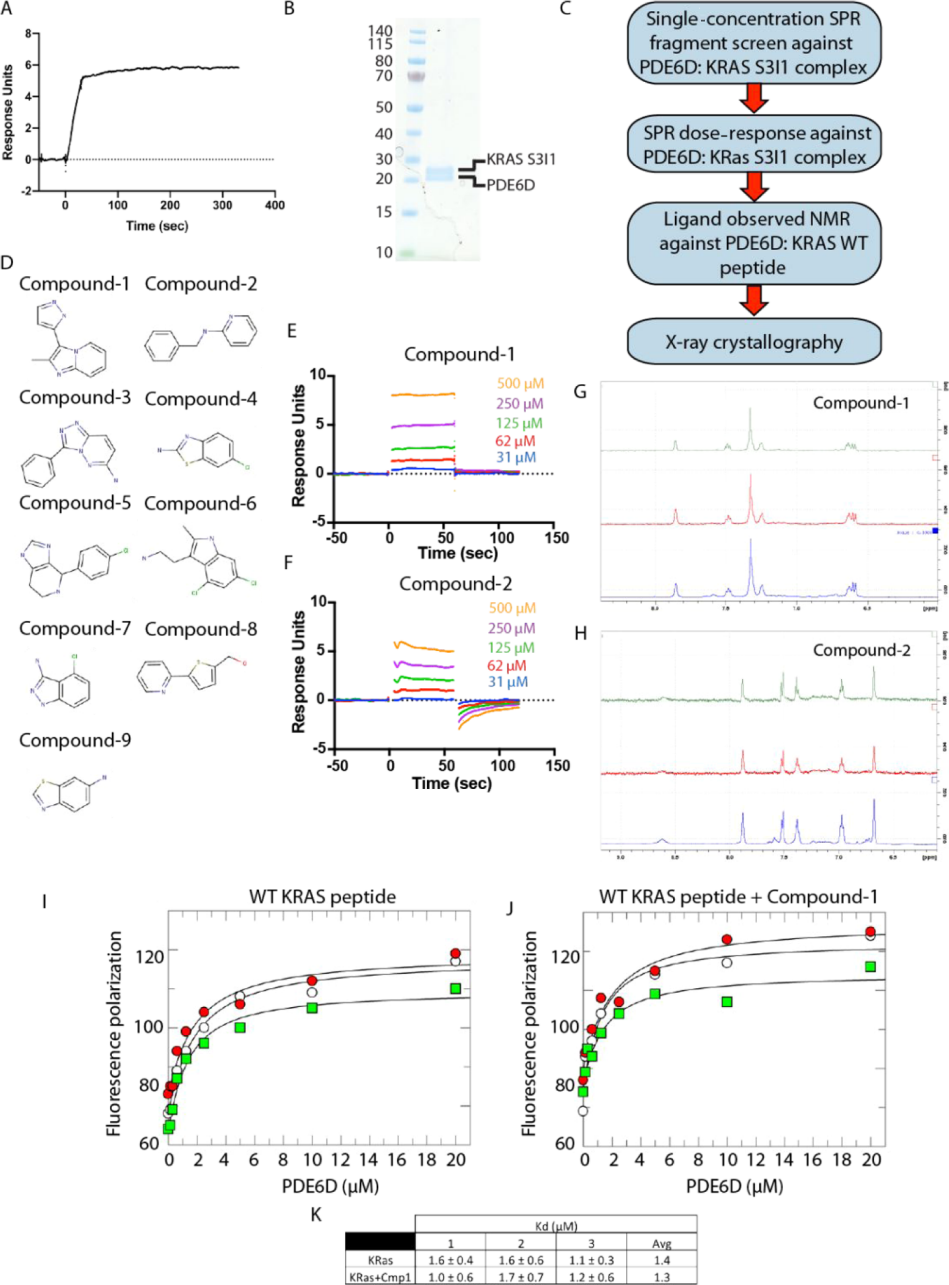
Identification of small-molecule fragments that bind to the PDE6D:KRAS complex. (A) SPR sensorgrams showing the binding of the high-affinity KRAS S3I1 mutant to immobilized PDE6D. Peptide (fluorescein-DGKKKKKKSSTIC(OMe)-Far) at 50 nM concentration. Image generated using Graphpad Prism 8. (B) SDS-PAGE gel showing a representative example of PDE6D:KRAS S3I1 complex purity used in the SPR fragment screen. (C) Schematic of the screening strategy employed. (D) Fragment hits obtained from the initial SPR fragment screen. (E,F) SPR dose–response for compound-1 and compound-2, respectively. (G) ^1^H NMR spectra of the aromatic region of compound-1 (0.5 mM) and PDE6D (10 μM). (Blue) ^1^H reference spectrum, (red) STD spectrum showing a good response from all the aromatic signals, and (green) STD with an additional KRAS WT HVR peptide (65 μM), showing a ∼30% decrease in the intensity, indicating a reduction in the affinity in the presence of the peptide. (H) ^1^H NMR spectra of the aromatic region of compound-2 (0.5 mM) and PDE6D (10 μM). STD spectrum with an additional KRAS WT HVR peptide (65 μM, green) showing no change in the ligand signal intensities, indicating no competition of the ligand with the peptide. (I) Triplicate titrations of PDE6D into the fluorescein-labeled KRAS HVR peptide (FAM-KKKKKKSKTKC(OMe)-Far) or (J) KRAS HVR peptide and compound-1 showing no loss in the affinity in the presence of compound-1. (K) Summary table of measured binding affinities from (H,I).

Having shown altered KRAS localization through the complex stabilization approach, we aimed to develop a screening strategy to identify fragments binding to the PDE6D:KRAS WT complex that could be developed into small-molecule stabilizers. It has been previously reported that the interaction between WT KRAS and PDE6D is transient with a fast off-rate^29^ and is consistent with the affinities in the micromolar range (see Figure S2A). This poses significant challenges when designing a high-throughput fragment-based screening method for SPR as the complex will simply dissociate before the screen can be performed. High-affinity binding, however, is often linked to a slow off-rate. As the KRAS S3I1 mutant binds to PDE6D with a very high affinity, which most likely indicates a slow off-rate, we sought to determine the off-rate of the complex using SPR. As seen in Figure 8A, the complex is remarkably stable with no measurable dissociation after a 6 min wash; Figure 8B shows the purity of the complex used in these assays. Exhibiting a very slow off-rate, the KRAS S3I1 variant enabled an SPR fragment screen to be performed against the complex. To ensure that all PDE6D was bound to full-length KRAS S3I1, two purification tags were used during purification of the complex—PDE6D contained two His_8_-tag and a KRAS S3I1 GST-tag which was subsequently removed following thrombin cleavage. The purified complex was subsequently immobilized onto a nitrilotriacetic acid (NTA) SPR chip using the double His_8_-tag on PDE6D before performing the fragment screen. To try and prevent the identification of fragments that favor the high-affinity mutant over KRAS(G12D) WT, a screening strategy was developed (Figure 8C), which enabled identification of fragments that could also bind to the PDE6D:KRAS WT complex.

An SPR hit was considered successful when a response of ≥5RU was recorded with fragments at a concentration of 500 μM, and a subsequent concentration dose–response was observed. Ligand-observed NMR was then employed to verify if the candidate compounds would not compete for PDE6D with the C-terminal prenylated tail of WT KRAS. In total, 9 fragments from a 915-fragment SPR screen were identified (Figure 8D), which were then used in cocrystallization studies to determine their binding modes. Two cocrystal structures with the fragments identified in the screen were successfully obtained: compound-1 in a ternary complex with PDE6D and WT KRAS peptide and compound-2 in complex with PDE6D alone. These two fragments interacted with the PDE6D:KRAS S3I1 complex with affinities in the low millimolar range (*K*_d_ > 0.5 mM, Figure 8E,F). To ensure that the compounds can bind to the KRAS WT:PDE6D complex, saturated transfer difference (STD) spectrum (Figure 8G,H) and KRAS WT peptide PDE6D affinity measurements in the absence or presence of the compound (Figures 8I–K and S6) were performed. Compound-1 showed no competition with KRAS in either experiment; however, compound-2 did show competition with the KRAS WT peptide.

### Structural Characterization of Fragment Binding Identifies Two Distinct Binding Sites

**Figure 9:**
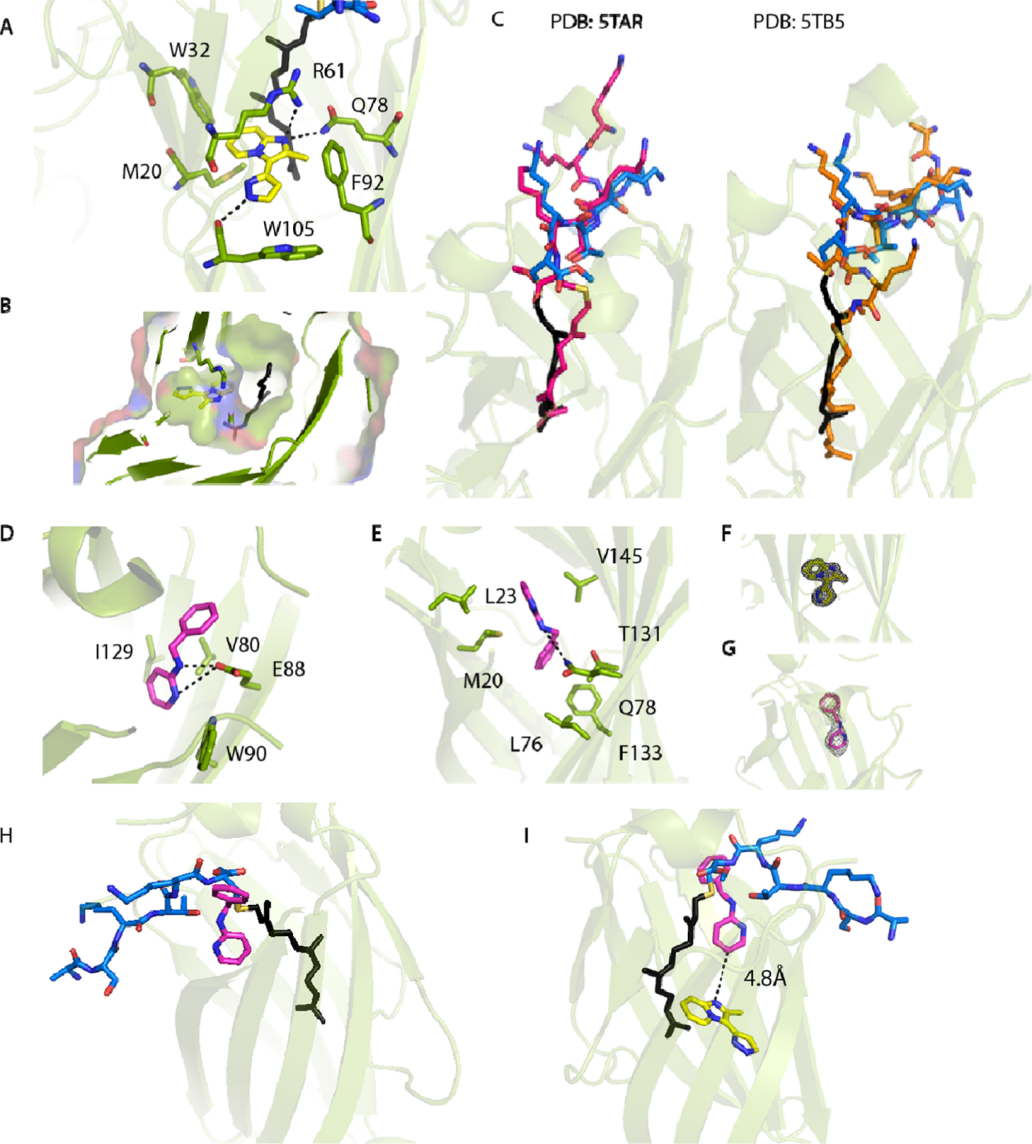
Characterization of compound-1 and compound-2 binding interactions with KRAS. (A) Binding site of compound-1 within the PDE6D:KRAS WT complex (PDB: 7Q9S). Compound-1 is shown in a stick form in yellow, PDE6D in green, the KRAS peptide in blue, and the KRAS farnesyl group in black. Hydrogen bonds are depicted as black dashes. (B) Surface representation of compound-1 bound to the PDE6D:KRAS complex with the surface shown at 40% transparency (PDB: 7Q9S). (C) Comparison of the peptide conformation in the presence of compound-1. The conformation of the KRAS peptide in the presence of compound-1 is shown in a blue stick form and overlaid with two existing PDE6D:KRAS structures (PDB:5TAR and PDB: 5TB5). (D) First binding site of compound-2 (PDB: 7QJK). Compound-2 shown in pink and PDE6D in green. Hydrogen bonds depicted as black dashes. (E) Second binding site of compound-2 to PDE6D deep within the PDE6D cargo-binding pocket (PDB:7QJK). Compound-2 is shown in a magenta stick form and PDE6D in green. Potential hydrogen bonds depicted as black dashes. (F,G) Electron density for compound-1 and compound-2 is shown in a black wire mesh form at 1σ. (H) Overlay of compound-2 (magenta) bound at the entrance of the PDE6D-binding pocket (PDB: 7QJK) with the KRAS WT peptide (blue and black) from the compound-1 structure (PDB: 7Q9S) (A), showing minimal steric clashes. (I) Binding sites of compound-1 and compound-2 are 4.8 Å apart, suggesting that it may be possible to link the two fragments.

In the crystal structure of PDE6D:KRAS peptide:compound-1 (Figure 9A,B), the asymmetric unit contains two copies of the ternary complex. In each copy, the KRAS peptide is present in a unique conformation, a phenomenon which has not been previously observed. In one of the two ternary complexes, the lipid-modified cysteine undergoes a significant conformational change so that when compared to the existing PDE6D:KRAS complex structures (PDB: 5TAR and 5TB5), it faces in the opposite direction; however, the rest of the peptide conformation remains largely conserved (Figure 9C).

To our surprise, the fragment is located deep within the PDE6D cargo-binding pocket at the interface of KRAS and PDE6D, binding adjacent to the farnesyl group of KRAS (Figure 9A,B). In one copy of the ternary complex, the compound is at 100% occupancy, while in the second ternary complex, it is modeled at 50% occupancy. With only 50% occupancy, several side chains within the PDE6D-binding pocket are in multiple conformations. In both instances, the fragment forms hydrogen bond interactions with the PDE6D R61 guanidine group, Q78 side chain, and carbonyl oxygen of the W105 peptide bond (Figure 9A).

In the crystal structure of the PDE6D:compound-2 complex, four copies of PDE6D are present in the asymmetric unit. This fragment, similarly to compound-1, also binds within the PDE6D cargo-binding pocket but occupies two distinct binding sites. One binding site, normally occupied by the side chain of W90 (Figure S7), is located at the entrance of the PDE6D-binding pocket (Figure 9D), where the fragment forms a hydrogen bond interaction with E88 and packs against W90 and V80. The second binding site is deep within the PDE6D-binding pocket, where compound-2 forms hydrophobic interactions with residues M20, L23, L38, L76, T131, and V145, as well as a weak hydrogen bond with Q78 (Figure 9E). Comparison of the PDE6D-binding pocket in PDE6D:compound-2 complex and the ternary complex containing compound-1 demonstrates that compound-2 forms limited steric clashes with the KRAS C-terminal cysteine residue when bound at the entrance of the PDE6D-binding pocket (Figure 9H). With significant conformational flexibility already observed in the context of KRAS binding to PDE6D (Figure S8), it is possible that an altered conformation is adopted in the presence of compound-2 to potentially accommodate its binding. The compound-2-binding site deep within the PDE6D cargo pocket does however overlap with the farnesyl group of KRAS and will compete with KRAS binding. Finally, we cannot exclude the possibility that compound-2 binds at a different site or with an altered conformation when KRAS is also present within the binding pocket.

As both fragments bind within the PDE6D-binding pocket and given that potential double-geranylgeranylated PDE6D cargo proteins were identified (Figure 2), we speculate that if PDE6D can bind double-geranylgeranylated cargo proteins, then our fragments may be interacting with the second lipid-binding site (modeled in Figure S9).

In the fragment-based drug discovery approach, it is generally advantageous to identify multiple fragments occupying different sites on the target protein. Such fragments can be subsequently linked to develop a compound with high affinity and specificity. Overlay of the two complexes containing compound-1 and compound-2 shows that these fragments are within close proximity to each other with a distance of 4.8 Å between the two closest atoms (Figure 9I). This implies the possibility to synthesize chimeric compounds comprising both fragments, with potentially improved affinity and specificity for PDE6D.

### HRAS Cysteine Residues Are Solvent-Exposed When Bound to PDE6D, Allowing for the Development of Irreversible Complex Stabilizers

**Figure 10:**
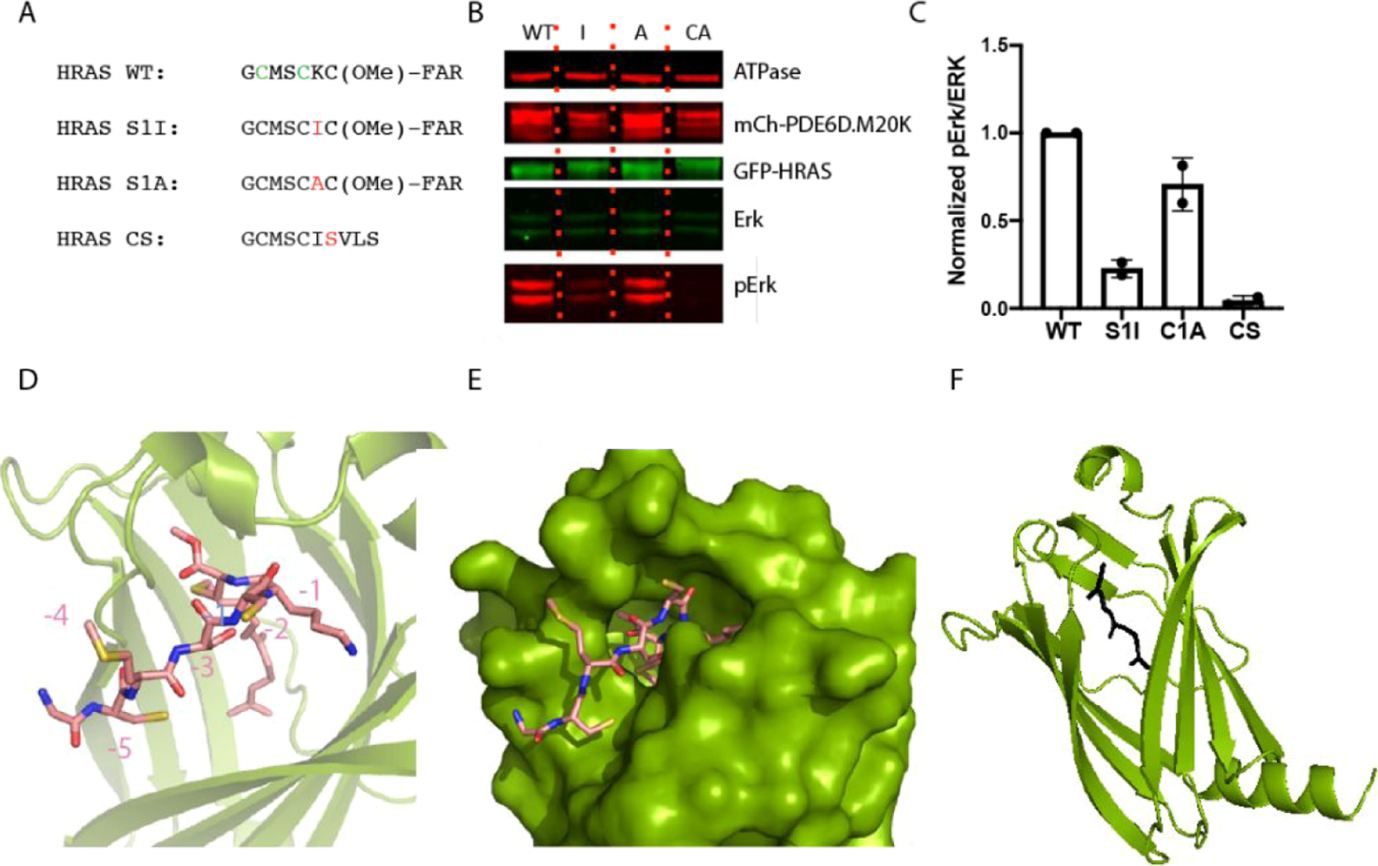
HRAS cysteine residues are solvent-exposed and could be targeted to obtain irreversible stabilizers. (A) Sequences of the HRAS C terminus with residue substitutions utilized in this study highlighted in red. Potential cysteine residues to be targeted for an irreversible binder are shown in green. (B) The high-affinity mutation results in a reduction of phospho-Erk levels; results quantified in (C). Comparable results were obtained from two independent experiments. (D) The crystal structure of HRAS bound to PDE6D has the peptide bound with residue numbering in chain with peptide residues modeled (PDB: 7QF9). (E) The cysteine residue of HRAS is solvent-exposed when bound to PDE6D and may therefore be targetable with irreversible compounds (PDB: 7QF9). (F) Crystal structure of the NRAS peptide bound to PDE6D (PDB: 7Q9R). Only the electron density for the farnesyl could be confidently built. The farnesyl moiety is shown in a black stick form and PDE6D in green cartoon.

As shown in Figure 2, both HRAS and NRAS copurify with PDE6D. By targeting the interface of the PDE6D:RAS complex, fragments that bind in proximity of the HVR of RAS could be used to leverage the sequence heterogeneity of the HVR to develop RAS isoform-specific inhibitors. Within their HVR sequences, both HRAS and NRAS contain a cysteine residue in the −5 position relative to the modified C-terminal cysteine (Figure 2C). HRAS has an additional cysteine residue in the −2 position. When HRAS and NRAS are bound to the plasma membrane, the cysteine residues are likely palmitoylated.^32^ Palmitoylation, however, is a reversible reaction, and the peptides identified in our PDE6D copurification were not lipid-modified. We therefore hypothesized that these cysteine residues could be targeted to irreversibly attach an inhibitor to both HRAS and NRAS, generating RAS isoform-specific PDE6D stabilizers. To ensure that enhanced binding to PDE6D can alter HRAS signaling, HRAS was mutated to conform to the high-affinity binding motif (Figure 10A). As HRAS naturally contains a serine residue in the −3 position, only the −1 residue needed to be mutated from a lysine to isoleucine to conform to the high-affinity PDE6D-binding motif. The levels of phosphorylated Erk were then compared against negative (alanine mutant) and positive (CAAX mutant) controls (Figure 10B). The high-affinity mutation results in a reduction in phospho-Erk levels when compared to the G12D WT and negative control, indicating that increasing the affinity of binding to PDE6D can be utilized by multiple RAS isoforms to alter signaling.

We next sought to identify the position and orientation of the cysteine residues in both HRAS and NRAS HVRs when bound to PDE6D to determine if they could be targeted with a chemical warhead. We were successfully able to cocrystallize PDE6D with C-terminally modified peptides of both HRAS and NRAS (Figure 10D–F).

The crystal structure containing HRAS peptide contains two copies of the complex, each in a different conformation (Figure 10D). In one complex, the density is readily interpreted for six amino acids including both cysteine residues, while in the second PDE6D:HRAS complex, only the carboxymethylated farnesylated C-terminal cysteine can be confidently modeled. As the electron density for only one copy of the HRAS peptide can be modeled, it suggests that HRAS, like KRAS, has conformational heterogeneity when bound to PDE6D. The cysteine residue in the −5 position is packed against PDE6D and not solvent-exposed (Figure S10)—whether this would occur with the full-length HRAS or this result is due to the potential increase in flexibility with a peptide is uncertain. As the −2 cysteine is solvent-exposed, it is possible that it could be targeted with an irreversible small molecule.

In the crystal structure of the NRAS HVR bound to PDE6D (Figure 10F), the electron density for only the farnesyl can be interpreted. This is most likely due to the flexibility of the peptide, whose composition, including a proline in the −1 and glycine in the −3 position relative to the lipidated cysteine, could provide unique conformational restraints compared to the other RAS isoforms. As NRAS has a cysteine in the −5 position, analogously to HRAS, it is likely that the cysteine would be in a similar conformation. Representative electron densities for all the prenylated peptides used in cocrystallization in this study are shown in Figure S9.

In summary, HRAS contains two cysteine residues within the HVR, of which at least one (and possibly both) is solvent-exposed and so could be targeted to develop an irreversible inhibitor.

## Discussion and Conclusions

The clinical importance of RAS in cancer necessitates the development of therapeutic strategies to target this recalcitrant protein. Despite decades of effort, only one direct RAS inhibitor has been approved for the treatment of RAS-driven cancers.^8,9^ Due to its combined importance and challenging nature, RAS has developed a reputation as the holy grail of cancer drug discovery. Here, we present a novel strategy for targeting RAS, sequestering it away from the plasma membrane by stabilizing its interaction with PDE6D. Using a rationally engineered, high-affinity KRAS mutant construct, we were successfully able to stabilize binding to PDE6D and inhibit release from PDE6D by the release factors ARL2 and ARL3. This increase in binding affinity successfully shifted the equilibrium of RAS localization, away from the plasma membrane, which resulted in reduced pErk signaling. Importantly, we also show that in a range of cell lines, there is a comparable expression level between PDE6D and KRAS, with some cell lines having a fivefold excess of PDE6D relative to KRAS, suggesting that even if all KRAS bound to PDE6D, there is likely to still be enough PDE6D to facilitate the correct trafficking of other prenylated proteins, limiting off-target effects. In cells where there is an excess of KRAS relative to PDE6D, this strategy will, however, be less effective; however, this is to be further investigated.

Utilizing the high-affinity mutant, we were able to develop an SPR fragment screening strategy against the complex and successfully identified fragments capable of binding to the complex. The fragments bind at the interface of PDE6D and KRAS and may serve as a starting point for fragment optimization. As we were successfully able to detect interactions between PDE6D and KRAS, as well as NRAS and HRAS, a potential advantage of the stabilization strategy would be to take advantage of the sequence heterogeneity within the HVR to develop isoform-specific stabilizers. While our fragment hits were bound in a surprising location—within the PDE6D cargo-binding pocket—they nevertheless bound at the PDE6D:KRAS interface—although away from the HVR residues—showing that there are as yet unexplored therapeutic strategies to target RAS.

Surprisingly, we did not detect PDE6D binding to KRAS4A; however, as no consensus PDE6D-binding motif beyond prenylation was identified, it is possible that PDE6D would be able to bind to and facilitate KRAS4A trafficking. As the role of KRAS4A in cancer becomes more apparent,^36^ strategies to specifically target this KRAS isoform will be needed to be identified, and the PDE6D stabilization strategy may present this possibility.

Given the novelty of this strategy, it could be interesting to try alternative approaches to maximize the chance of success in identifying compounds which bind at the surface of PDE6D and directly stabilize the complex in proximity of the HVR residues. These include, for example, a FRET-based assay with more drug-like compounds, or a screen using an irreversible compound library to directly target the cysteine residues on HRAS, rather than the fragment based approach we utilized in this study.

Finally, our PDE6D copurification assays identified double-geranylgeranylated interactors, and the observed binding of fragments within the PDE6D-binding pocket—even in the presence of the KRAS—indicates that the binding pocket may provide sufficient space to accommodate such large lipid modifications. Unfortunately, due to the expense and technical challenge of synthesizing double-geranylgeranylated carboxymethylated peptides, we were unable to biophysically validate this possibility. Based on these results, we postulate that PDE6D could be involved in trafficking double-geranylgeranylated protein cargos, therefore playing a more generic and not yet fully explored role in trafficking prenylated proteins.

## Materials and Methods

### Cloning

All constructs and their associated primers are provided in the Supporting Information—Table S1.

A TEV-cleavable His_12_-MBP-tagged KRAS WT construct was purchased as a codon-optimized g-block from IDT, cloned into a pCDNA 3.1-C-His plasmid, and used as a template for all MBP-tagged mutations.

PDE6D was cloned into pRSF-Duet and pBDDB-SPR3 vectors for SPR experiments.^37^

All PCRs were performed using Q5 polymerase (NEB) following the manufacturer’s guidelines. All mutagenic primers were designed and annealing temperatures determined using the Q5 BaseChanger (https://nebasechanger.neb.com/) website. The primers were purchased from IDT.

### Cell Culture

NIH-3T3 fibroblasts (CRL-1658) and Daoy (HTB-186) were obtained from the ATCC (Manassas, Virginia, US) and cultured following the provider’s guidelines using Dulbecco’s modified Eagle medium (DMEM) (Gibco, Thermo Fisher Scientific Inc., Waltham, MA, USA), 10% calf serum (ATCC), and Eagle’s minimum essential medium supplemented with 10% fetal serum. RPE (ATCC, CRL-4000, human, female), A549, and H2009 were maintained in DMEM:F12 (Thermo Fisher Scientific). H358 cells were cultured in RPMI 1640 (Thermo Fisher Scientific). Jurkat clone E6-1 (TIB-152, human male) were grown in RMPI 1640 supplemented with 10% inactivated fetal bovine serum (Sigma), 1 mM sodium pyruvate, and 10 mM HEPES (both from Sigma). HEK-293FT and human primary fibroblasts were maintained in DMEM. All media were supplemented with 2 mM l-glutamine and 100 units/mL of penicillin–0.1 mg/mL streptomycin (Sigma, Merck KGaA, Darmstadt, Germany), and unless stated otherwise, with 10% fetal serum. All cell lines were grown at 37 °C and 5% CO_2_.

### Immunofluorescence

The NIH-3T3 cells were transfected with KRAS and PDE6D constructs using Lipofectamine 2000 following the provider’s guidelines. After transfection, the cells were incubated overnight for 24 h and transferred onto 1.5-thickness glass coverslips (VWR, Avantor, Radnor, PA, US) and allowed to attach overnight. Next, the cells were starved with 0.5% calf serum-containing media for 24 h and fixed with 4% paraformaldehyde (Thermo Fisher Scientific) for 20 min. After fixation, the coverslips were extensively washed with phosphate-buffered saline (PBS) and the cells were permeabilized using 0.3% Triton X-100 (Sigma/Merck) for 5 min at room temperature. After washing in PBS, the samples were blocked using 2% bovine serum albumin (BSA) (Sigma/Merck) for at least 30 min, followed by antiacetylated tubulin MABT868 (Sigma) overnight at 4 °C. After extensive washing in PBS, the cells were incubated with goat antimouse IgG H + L antibody Abberior Star Red from Abberior (GmbH, Göttingen, Germany) for 45 min in darkness at room temperature. The coverslips were mounted onto glass slides using fluoromount-G (Southern Biotech, Birmingham, AL, USA) and cured for at least 6 h before imaging.

### Confocal Microscopy and Colocalization Analyses

Images were acquired using a Zeiss 710 upright confocal microscope (Zeiss, Jena, Germany) equipped with a plan-apochromat 63×/1.4 oil DIC M27 objective lens. Three serial *z*-stack images were acquired for each field of view using a 1 μm optical slice for every channel, with a *z*-step of 0.5 μm. All microscopy files were processed using Fiji open source software.^38^ To examine the ciliary localization of KRAS, ROIs of 5 μm^2^ containing cilia were drawn in cells GFP+ (overexpressing KRAS) or GFP+ and mCherry+ (overexpressing both KRAS and PDE6D). We performed colocalization analyses in these ROIs using the built-in Fiji plug-in “Coloc 2” obtaining the Pearson coefficient for KRAS (GFP) versus acetylated tubulin.

### Peptides

The KRAS S3I1 peptide [sequence: fluorescein-DGKKKKKKSSTIC(OMe)-farnesyl], KRAS WT [peptide sequence: DGKKKKKSKTKC(OMe)-farnesyl], and HRAS [peptide sequence: ESGPGCMSCKC(OMe)-farnesyl] were synthesized by JPT Innovative Peptide Solutions. Cys(OMe)-geranylgeranyl was synthesized by Medicilon-Shanghai, and Cys(OMe)-farnesyl was purchased from Abcam. NRAS peptide sequence GTQGCMGLPC(OMe)-farnesyl was synthesized using a protocol adapted from previously published protocols.^39^

### Protein Purification

#### PDE6D

Following a fresh transformation into BL21 DE3 cells, the cultures were inoculated and grown at 37 °C until the O.D._600nm_ reached ∼0.5. The cells were cooled to 20 °C, induced with 0.2 mM isopropyl β-D-1-thiogalactopyranoside (IPTG), and left to express overnight. The cells were harvested with centrifugation and resuspended in the lysis buffer (50 mM Tris pH 7.5, 300 mM NaCl, and 2 mM β-mercaptoethanol) and lysed using a microfluidizer at 20,000 psi. The lysed cells were centrifuged at 48,000*g*, filtered through a 5 μM filter, and loaded onto a 5 mL HisTrap column (Cytiva) at 3 mL min^–1^ using a P960 peristaltic pump. The column was washed in 40 mM imidazole before eluting with a linear gradient of 0–300 mM imidazole. PDE6D used to form the complex with KRAS was then passed through a Superdex S75 size-exclusion column equilibrated in 20 mM Tris pH 7.5, 150 mM NaCl, and 2 mM β-mercaptoethanol. PDE6D to be used for pulldown assays and fluorescence polarization was dialyzed overnight in 20 mM Tris pH 7.5, 150 mM NaCl, and 2 mM β-mercaptoethanol in the presence of TEV protease to remove the polyhistidine tag. The following day, the dialyzed protein was passed over a HisTrap column and the flow-through collected before passing over a Superdex S75 size-exclusion column equilibrated in 20 mM Tris pH 7.5 and 2 mM dithiothreitol (DTT). The purity was assessed on a SDS-PAGE gel concentrated to 20 mg mL^–1^ and snap-frozen in liquid nitrogen before storing at −80 °C.

#### GST-RAB1B

Following a fresh transformation into BL21 DE3 cells, the cultures were inoculated and grown at 37 °C until the O.D._600nm_ reached ∼0.5. The cells were cooled to 20 °C, induced with 0.2 mM IPTG, and left to express overnight. The cells were harvested with centrifugation and resuspended in the lysis buffer (50 mM Tris pH 7.5, 300 mM NaCl, 2 mM β-mercaptoethanol, and 5 mM MgCl_2_) and lysed using a microfluidizer at 20,000 psi. The lysed cells were centrifuged at 48,000*g*, filtered through a 5 μM filter, and loaded onto a 5 ml GSTrap column (Cytiva) at 3 mL min^–1^ using a P960 peristaltic pump. The column was washed with 50 ml of the lysis buffer before eluting with the lysis buffer supplemented with 10 mM glutathione. The eluted protein was subsequently passed through a Superdex S200 16/60 size-exclusion column equilibrated in 20 mM Tris pH 7.5, 150 mM NaCl, 1 mM DTT, and 5 mM MgCl_2_.

#### PDE6D:KRAS S3I1 Complex for SPR

The HEK293F cells were transfected at a density of 1.0–1.4 × 10^6^ cells per mL with 1.25 mg of the DNA GST-tagged KRAS S2I1 construct per liter transfected using polyethylenimine (PEI) as a transfection reagent. Four days post-transfection, the cells were harvested by centrifugation at 3000*g* for 5 min. The cells were suspended in the lysis buffer (40 mM Tris pH 7.5, 300 mM NaCl, 5 mM MgCl_2_, 2 mM β-mercaptoethanol, and protease inhibitors). 50+ mg of PDE6D SPR3 was added to the resuspended HEK293F cells, and lysis was performed using sonication. The lysates were subjected to nickel purification as outlined above. The eluted protein was loaded onto a 5 mL GSTrap column using a P960 peristaltic pump and washed with a buffer containing 20 mM Tris pH 7.5, 100 mM NaCl, 5 mM MgCl_2_, and 2 mM β-mercaptoethanol before cleaving off the GST-tag using thrombin overnight on the column. The following day, the cleaved protein was pooled and loaded onto a 1 mL HisTrap column using a P960 peristaltic pump and washed in 20 mM Tris pH 7.5, 150 mM NaCl, 5 mM MgCl_2_, and 2 mM β-mercaptoethanol before eluting with a linear gradient of imidazole from 0 to 500 mM. Fractions containing PDE6D and KRAS at a 1:1 ratio were assessed using SDS-PAGE gels and dialyzed overnight in a buffer containing 20 mM Tris pH 7.5, 150 mM NaCl, 5 mM MgCl_2_, and 2 mM DTT. The protein was used directly for SPR fragment screening following dialysis without freezing.

#### PDE6D KRAS S3I1 Complex for Crystallography

HEK293F cells were transfected at a density of 1.0–1.4 × 10^6^ cells per mL with 1.25 mg of DNA-untagged KRAS S3I1 construct per liter transfected using PEI as a transfection reagent. The cells were harvested, lysed, and nickel purification performed as per the PDE6D:KRAS S3I1 complex for SPR. Nickel elution fractions containing the PDE6D:KRAS S3I1 complex were pooled and dialyzed overnight in the presence of TEV protease in a buffer containing 20 mM Tris pH 7.5, 150 mM NaCl, 5 mM MgCl_2_, and 2 mM β-mercaptoethanol. The following day, the dialyzed protein was passed over a 5 mL HisTrap column using a P960 peristaltic pump and the flow-through collected, concentrated with a centrifugal concentrator, and injected onto a Superdex S75 size-exclusion column equilibrated in 20 mM Tris pH 7.5, 150 mM NaCl, 5 mM MgCl_2_, and 2 mM DTT. Fractions containing PDE6D and KRAS S3I1 at a 1:1 ratio were assessed using SDS-PAGE gels, pooled and concentrated to ∼10 mg mL^–1^, and flash-frozen in liquid nitrogen and stored at −80 °C.

#### MBP-KRAS Constructs for Pulldown and Release Assays

The HEK293F cells were transfected at a density of 1.0–1.4 × 10^6^ cells per mL with 1.25 mg of the DNA MBP-tagged KRAS construct per liter transfected using PEI as a transfection reagent. The cells were harvested, lysed, and nickel purification performed as per the PDE6D:KRAS S3I1 complex for SPR. The eluted protein was dialyzed into a buffer containing 20 mM MES pH 6.0, 100 mM NaCl, 5 mM MgCl_2_, and 2 mM DTT. Following dialysis, the protein was diluted in the dialysis buffer without NaCl at a 1:1 ratio. The protein was loaded using a peristaltic pump at 1 mL min^–1^ onto an ion exchange column and washed in 20 mM Tris pH 7.5, 50 mM NaCl, 5 mM MgCl_2_, and 2 mM DTT before eluting with a linear gradient of 0–700 mM NaCl. The fractions containing MBP-tagged KRAS were pooled and snap-frozen in liquid nitrogen.

#### ARL2 and ARL3 Purification and Nucleotide Exchange

ARL2 and ARL3 were purified and subjected to nucleotide exchange as described previously.^35^

### Identification of PDE6D Interactors

1 L of suspension freestyle 293F HEK cells (Thermo Fisher Scientific) was pelleted, lysed by sonication in 50 mM Tris 7.5, 300 mM NaCl, and 5 mM MgCl_2_, and supplemented with 2 mM β-mercaptoethanol and 20 mg of non-TEV-processed pBDDP-SPR3 PDE6D. The cell lysate was clarified with centrifugation and filtration before passing over a 5 mL NTA nickel column. The loaded column was washed with 60 mM imidazole before eluting with a linear gradient 0–300 mM. The eluted protein was pooled and dialyzed overnight at 4 °C against 20 mM Tris pH 7.5, 150 mM NaCl, 5 mM MgCl_2_, and 2 mM β-mercaptoethanol in the presence of TEV protease. The following day, the dialyzed protein was passed over nickel resin and the flow-through collected, concentrated, and passed through a Superdex 75 size-exclusion column in 20 mM Tris 7.5, 150 mM NaCl 5 mM MgCl_2_, and 2 mM DTT. Following elution, the fractions were run on a 4–12% SDS-PAGE gel. Protein bands other than PDE6D were excised and sent to AltaBioscience for proteomic mass spectrometry analysis.

### GST-RAB1B Pulldown

GST-RAB1B underwent nucleotide exchange following a published protocol.^40^ 20 μg of GST-RAB1B or 10 μg of GST was incubated with 20 μg of PDE6D in a buffer containing 20 mM Tris pH 7.5, 200 mM NaCl, 1 mM DTT, and 5 mM MgCl_2_. The protein was added to 50 μL of GST-resin and incubated at room temperature for 30 min, following which the resin was centrifuged at 1000*g* and the supernatant aspirated. Four washes were performed before eluting the protein in a buffer containing 20 mM Tris pH 7.5, 200 mM NaCl, 1 mM DTT, 5 mM MgCl_2_, and 10 mM glutathione. The eluted samples were run subsequently on an SDS-PAGE gel, and the subsequent western blot was probed using anti-GST (Santa Cruz sc-138) and anti-PDE6D (CSB-Cat:PA526126L) primary antibodies. The secondary antibodies used were Li-Cor Cat: 926-32212 and 926-68073, and images were generated using a Li-Cor instrument.

### Fluorescence Polarization Binding Assay

#### High-Affinity KRAS S3I1 Peptide Binding to PDE6D

Fluorescent polarization measurements were recorded using a TecanSpark plate reader using 96-well Corning black half-area plates using an excitation and emission wavelength of 485 and 530 nM, respectively. The measurements were recorded in a buffer containing 20 mM Tris pH 7.5, 150 mM NaCl, and 2 mM DTT. The fluorescein-labeled KRAS S3I1 peptide [sequence: fluorescein-DGKKKKKKSKTKC(OMe)-Far] was used at 5 nM, and the PDE6D concentration range was 0–512 nM.

#### WT G12D KRAS Peptide Binding to PDE6D with and without the Compound

The measurements were recorded in a buffer containing 20 mM Tris (pH 7.5), 150 mM NaCl, 2 mM DTT, and 5% DMSO. The KRAS peptide [fluorescein-KKKKKKSKTKC(OMe)-Far] was used at 500 nM with the compound at 500 μM. PDE6D was titrated with twofold dilutions from 20 to 0.156 μM.

#### Affinity Calculations

To obtain the dissociation constants, the fluorescence polarization binding data was fitted to a quadratic equation using GraFit: *F*_P_ = *F*_min_ – (*F*_min_ – *F*_max_) * (*E* + *L* + *K*_d-sqrt_[(*E* + *L* + *K*_d_)^2^ – 4*E*L)]/(2*E), where *F*_P_ is the fluorescence polarization, *F*_min_ and *F*_max_ are the minimum and maximum polarization signal, respectively, *L* is the protein concentration, *K*_d_ is the dissociation constant, and *E* is the constant—fluorescently labeled peptide concentration.

### MBP-KRAS A3A1 and MBP-KRAS I3S1 SPR Binding

Anti-MBP camelid monobodies (Chromotek) were immobilized onto a CM5 Series 5 (Cytiva) chip using the manufacturer’s protocol. All data was acquired using a Biacore T200 instrument. MBP-tagged KRAS constructs were immobilized at a flow rate of 10 μL min^–1^ prior to the experiment. PDE6D at concentrations of 0.04–20 μM with twofold serial dilutions were injected onto the chip. Data was analyzed using Biacore analysis software and figures generated using Graphpad Prism 8.

### Pulldown and Release Assays

Pulldown and release assay experiments were performed in a buffer containing 20 mM Tris pH 7.5, 200 mM NaCl, 5 mM MgCl_2,_ 2 mM DTT, and 100 μM GppNHp. 10 μg of MBP-KRAS was incubated with 10 μg of untagged PDE6D.M20K and either 20 μg of ARL2.GTP, ARL3.GTP, or equivalent volume of buffer, and the buffer was added to a total volume of 60 μL and left for 20 min at room temperature. 70 μL of amylose-bead slurry (NEB) was equilibrated in the wash buffer. 200 μL of the wash buffer was added to each reaction before adding to the amylose resin and left for 10 min with gentle agitation. The reactions were centrifuged at 1000*g* and the liquid aspirated. Resin was washed in buffer four times before eluting in the wash buffer supplemented with 10 mM maltose. The samples were immunoblotted and bands detected using the following antibodies: anti-His (Clontech-Cat: 631212), anti-PDE6D (CSB-Cat: PA526126L), and anti-KRAS (Abcam-Cat: ab-180772). The secondary antibodies used were Li-Cor Cat: 926-32212 and 926-68073, and images were generated using a Li-Cor instrument.

### pErk/Erk and Membrane Fractionation

Freestyle HEK293F cells were transfected with a 50:50 mixture of mCherry-PDE6D.M20K and GFP-KRAS using PEI. The following day, the transfection cells were harvested at 3000*g* for 3 min before resuspending the cells in 20 mM Tris pH 7.5, 120 mM NaCl, 5 mM MgCl_2_, and 2 mM DTT and lysis by sonication. The samples for pErk/Erk western blot were taken for immunoblotting. The samples for membrane fractionation were spun at 100,000*g* for 1 h. The soluble fraction was decanted and the pellet resuspended using a Dounce homogenizer in an equal volume as the soluble fraction. Samples of both soluble and membrane fractions were run on a 4–12% SAS-PAGE gel and western blots performed. The antibodies used here were anti-GFP (Santa Cruz Cat: SC-9996), anti-Erk (Cell Signaling Cat: 4696S), anti-pErk (Cell Signaling Cat: 9101S), anti-sodium/potassium ATPase (Abcam Cat: AB76020), anti-mCherry (Abcam Cat: AB6745), and anti-GAPDH (Santa Cruz Cat: SC-47724). The secondary antibodies used were Li-Cor Cat: 926-32212 and 926-68073, and images were generated using a Li-Cor instrument.

### SPR KRAS S3I1 PDE6D SPR Binding Stability Assay

The experiment was performed using a Biacore T200 at 25 °C. PDE6D SPR3 at 5 μg mL^–1^ was loaded onto a Series S NTA (Cytiva) chip at 10 μL min^–1^ until ∼600 RU was recorded following 1 min surface activation with 0.5 mM NiCl_2_. The fluorescently labeled KRAS S3I1 peptide was injected at a concentration of 50 nM for 30 s before washing the chip for 6 min in the buffer (20 mM Tris pH 7.5, 150 mM NaCl, and 5 mM β-mercaptoethanol).

### SPR Fragment Screening

Initial fragment screening was performed using a Biacore 4K instrument at 25 °C. The buffer contained 20 mM Tris pH 7.4, 150 mM NaCl, 1 mM tris(2-carboxyethyl)phosphine, 5 mM MgCl_2_, 0.05% P20, and 5% DMSO. A 915-compound library was screened at a single concentration of 500 μM against the PDE6D:KRAS S3I1 complex immobilized (concentration at injection: 200 nM) onto an NTA chip with at least 4000 RU immobilized after 1 min of surface activation with 0.5 mM NiCl_2_. Potential hits (>5 RU) were repeated on a T200 Biacore instrument with twofold serial dilutions from 500 to 31 μM. A solvent correction was performed for all experiments. Data was analyzed using BIAevaluation/Insight software. The figures were generated using Graphpad Prism 8.

### Ligand-Observed NMR Binding Experiments

All NMR data were recorded at 300 K on a Bruker AVANCE NEO 600 spectrometer operating at 599.782 MHz using a 5 mm QCI-F cryoprobe. For each sample, the proton (^1^H) and the saturation transfer difference (STD) spectra with a 2 s selective saturation pulse at 0.78 ppm were recorded. The NMR samples were prepared with 10 μM PDE6D and a 500 μM ligand in 30 mM Tris, 50 mM NaCl, and 5 mM MgCl_2_ at pH 7.5 containing 90/5/5% H_2_O/D_2_O/DMSO. The 65 μM KRAS WT HVR peptide was added for the competition experiments. As reference, the experiments were also performed using the fragment alone to exclude artifacts. Data was analyzed using ACD/Lab and Topspin software.

### Isothermal Titration Calorimetry

All ITC titrations were performed at 25 °C with a reference power of 6 DP using a PEAQ-ITC200 instrument (Malvern). All titrations were in 10 mM Tris pH 7.5, 150 mM NaCl, 2 mM DTT, and 5% DMSO. PDE6D was loaded into the syringe at a concentration between 100 and 300 μM. The peptides were loaded into the sample cell at concentrations between 10 and 30 μM. Following an initial injection of 0.4 μL, 19 subsequent injections of 2.0 μL were performed. Following the subtraction of control titrations (PDE6D:buffer), the data was analyzed with PEAQ analysis software.

### Crystallization

Sparse matrix crystallization screens were performed in 96-well plates using a Mosquito (SPT Labtech) for drop dispensing. PDE6D:KRAS S3I1 complex crystals were obtained under a condition with 0.2 M AmSO_4_, 0.1 M trisodium citrate of pH 5.6, and 15% (w/v) PEG 4000 with the complex at 8 mg mL^–1^ at 291 K. The crystals were cryoprotected in the reservoir solution supplemented with 25% ethylene glycol. PDE6D:KRAS peptide:compound-1 crystals were obtained under a condition with 0.2 M lithium sulfate, 0.1 M Tris of pH 8.5, and 20% PEG 3000 at 293 K with 10 mg mL^–1^ PDE6D, 1 mM KRAS peptide, and 8 mM compound-1. The crystals were flash-frozen in liquid nitrogen in the reservoir solution supplemented with 25% ethylene glycol. PDE6D:compound-2 crystals were obtained under a condition with 1.2 M AmSO_4_ and 0.1 M ammonium acetate of pH 4.9 at 291 K. PDE6D at 20 mg mL^–1^ was mixed with compound-2 to produce a final concentration of the compound of 8 mM. The crystals were cryoprotected in the reservoir solution supplemented with 10% ethylene glycol and 15% glycerol. HRAS peptide PDE6D complex crystals were obtained under a condition with 0.1 M Tris of pH 8.5 and 8% PEG 8000. The crystals were grown at 291 K with PDE6D at 10 mg mL^–1^ and 0.7 mM HRAS peptide. The crystals were cryoprotected with the reservoir solution supplemented with 25% ethylene glycol. NRAS peptide PDE6D complex crystals were obtained at 16 mg mL^–1^ protein and 1 mM peptide under a condition with 0.1 M citric acid of pH 4.0 and 3.2 M AmSO_4_ at 279 K. The crystals were cryoprotected in the reservoir solution supplemented with 25% glycerol and flash-frozen in liquid nitrogen. PDE6D in complex with Cys(OMe)-geranylgeranyl was obtained under the Qiagen screen PEGs II condition B7 at 277 K with PDE6D at 10 mg mL^–1^ and the modified cysteine at 1 mM. The crystals were cryoprotected in the reservoir solution supplemented with 25% ethylene glycol.

### Structure Determination and Refinement

With the exception of the PDE:Cys(OMe)-Ger complex, which was collected at the Swiss Light Source, all data sets were obtained at the Diamond Light Source beam lines I03, I04, and I04-1. Initial data processing was performed using either Xia2^41^ or Dials,^42^ except the compound-2 data set which was processed with XDS^43^ and Aimless.^44^ All structures were phased using molecular replacement using Phaser^45^ of the CCP4 program suite.^46^ All structures containing PDE6D were phased using the PDE6D structure from PDB: 3T5G as a search model. The KRAS S3I1 component of the PDE6D:KRAS S3I1 complex was phased using the KRAS structure from PDB: 5TAR. Following molecular replacement, the structures were refined using iterative cycles of the manual model building in Coot^47^ and using REFMAC5.^48^ The PDB and CIF files for compound-1 and -2 were generated using JLigand^49^ and PRODRG.^50^

## Supporting Information Available

GST-RAB1B pulldown with PDE6D, prenylated carboxymethylated cysteine as the sole determinant for PDE6D binding, binding of MBP-KRAS A3A1 and MBP-KRAS I3A1 to PDE6D, comparison between KRAS S3I1 and INPP5E conformation when bound to PDE6D, KRAS S3I1 mutant is the membrane-binding competent, compound-2 effect on KRAS binding to PDE6D, compound-2 occupying a site that PDE6D residue W90 can occupy, KRAS C-terminal residues having significant conformational heterogeneity when bound to PDE6D, overlay of double-geranylgeranylated cysteine, HRAS cysteine in position −5 that is not solvent-exposed, electron density for bound peptides, and crystallographic table of statistics (PDF)

## Notes

PDB IDs of new crystal structures: PDE6D:KRAS S3I1 complex (7Q9U), PDE6D:HRAS peptide complex (7QF9), PDE6D:NRAS peptide complex (7Q9R), PDE6D:KRAS peptide:compound-1 (7Q9S), PDE6D:compound-2 complex (7QJK) and PDE6D:Cys(OMe)-geranylgeranyl (7Q9Q).

## Notes

Authors will release the atomic coordinates upon article publication.

## Abbreviations

ARL2: ADP ribosylation factor-like GTPase 2
ARL3: ADP ribosylation factor -like GTPase 3
ARF5: ADP-ribosylation factor 5
CAAX: (motif) cysteine residue followed by two aliphatic residues and any C-terminal residue
FTase: farnesyltransferase
GAP: GTPase-activating protein
GEF: guanine nucleotide exchange factor
HVR: hypervariable region
INPP5E: inositol polyphosphate-5-phosphatase E
MBP: myelin basic protein
PDE6D: phosphodiesterase 6 delta
RAB1B: RAS-related protein 1B
RheB: RAS homolog enriched in brain
ROI: region of interest
RPE: retinal pigment epithelial cells
SPR: surface plasmon resonance
STD: saturated transfer difference
