# Supplementary material for: Stabilization of the RAS:PDE6D Complex Is a Novel Strategy to Inhibit RAS Signaling
Source: J Med Chem. 2022 Feb 2;65(3):1898–914. doi: 10.1021/acs.jmedchem.1c01265 (PMC8842248; doi:10.1021/acs.jmedchem.1c01265)
Supplement: Supplementary file 1 — jm1c01265_si_001.pdf [file jm1c01265_si_001.pdf]

Supporting Information

Stabilisation of the Ras:PDE6D complex is a novel strategy to inhibit Ras signalling

Tamas Yelland[1+], Esther Garcia[1+], Charles Parry[2], Dominika Kowalczyk[1], Marta Wojnowska[3], Andrea Gohlke[2,], Matja Zalar[2,4], Kenneth Cameron[2], Gillian Goodwin[2,5], Yu Qing[6], Zhu Peng-Cheng[6], Yasmin ElMaghloob[1], Angelo Pugliese[2,5], Lewis Archibald[7], Andrew Jamieson[7], Chen Yong Xiang[6], Duncan McArthur[2,5], Justin Bower[2*], Shehab Ismail[1,8,*]

[1] CRUK Beatson Institute, Glasgow, United Kingdom, G61 1BD
[2] Drug Discovery Program, CRUK Beatson Institute, Glasgow, United Kingdom, G61 1BD
[3] School of Chemistry, North Haugh, University of St Andrews, KY16 9ST St Andrews, United Kingdom
[4]School of Chemical Engineering and Analytical Sciences, Faculty of Science and Engineering, University of Manchester, Manchester, M13 9PL, United Kingdom
[5]BioAscent Discovery Ltd, Biocity, Motherwell, United Kingdom, ML1 5UH
[6] Key Laboratory of Bioorganic Phosphorus Chemistry and Chemical Biology, Department of Chemistry, Tsinghua University, China
[7] School of Chemistry, University of Glasgow, Glasgow, United Kingdom, G12 8QQ
[8]Present address: Department of Chemistry, KU Leuven, Celestijnenlaan 200G, 3001 Heverlee, Belgium
+ these authors contributed equally
* Lead contact j.bower@beatson.gla.ac.uk, shehab.ismail@kuleuven.be

Contents of Supplementary Information:

Supplementary Figure 1. GST-RAB1B pulldown with PDE6D.
Supplementary Figure 2. Prenylated carboxymethylated cysteine is the sole determinant for PDE6D binding.
Supplementary Figure 3. Binding of MBP-KRAS A3A1 and MBP-KRAS I3A1 to PDE6D.
Supplementary Figure 4. Comparison between KRAS S3I1 and INPP5E conformation when bound to PDE6D.
Supplementary Figure 5. The KRAS S3I1 mutant is membrane binding competent.
Figure 6. Compound-2 affect of KRAS binding to PDE6D.
Supplementary Figure 7. Compound-2 occupies a site that PDE6D residue W90 can occupy.
Supplementary Figure 8. KRAS C-terminal residues have significant conformational heterogeneity when bound to PDE6D.
Supplementary Figure 9.
Supplementary Figure 10. The HRAS cysteine in position -5 is not solvent exposed.
Supplementary Figure 11. Electron density for bound peptides.
Table 1: Crystallographic table of statistics
List of constructs.

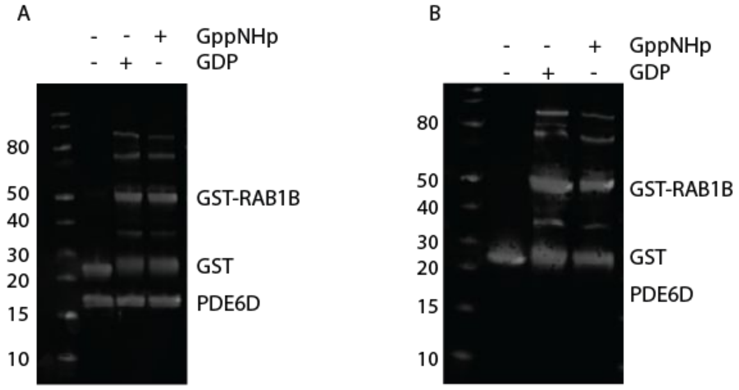

Figure S1. GST-RAB1B pulldown with PDE6D. A. Input for pulldown of GST-tagged-RAB1B with PDE6D. B. Elute of pulldown showing no interaction with PDE6D, which occurs independently of RAB1B nucleotide bound state.

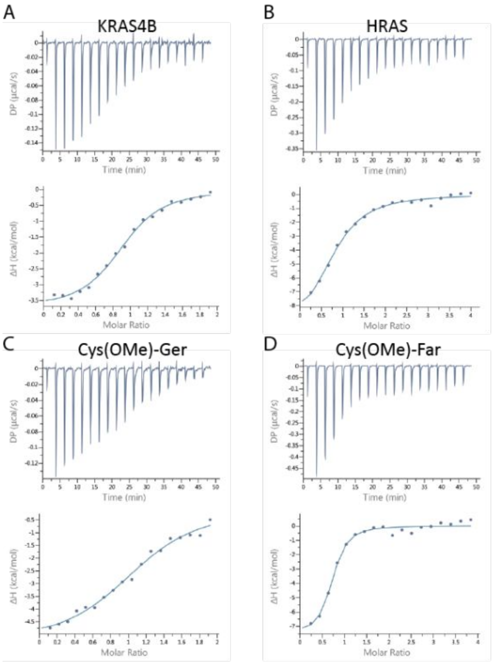

E

Peptide	Kd(µM)
KRAS	1.38±0.27
HRAS	2.67±0.46
Cys(OMe)-Far	0.9±0.29
Cys(OMe)-Ger	2.73±0.90

F

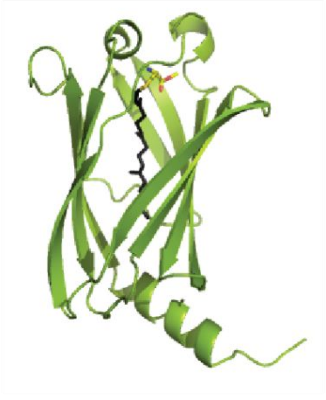

Figure S2. Prenylated carboxymethylated cysteine is the sole determinant for PDE6D binding. A ITC binding curves for KRAS peptide (DGKKKKKKSKTKC(OMe)-Far to PDE6D. B. ITC binding curves for HRAS peptide (PDESGPGCMSCKC(OMe)-Far to PDE6D. C. ITC binding curves for Cys(OMe)-Ger to PDE6D. D. ITC binding curves for Cys(OMe)-Far to PDE6D. E. Table of measured affinities of each peptide to PDE6D. F. Crystal structure of Cys(OMe)-Ger to PDE6D with PDE6D in green and Cys(OMe)-Ger in black and yellow stick form.

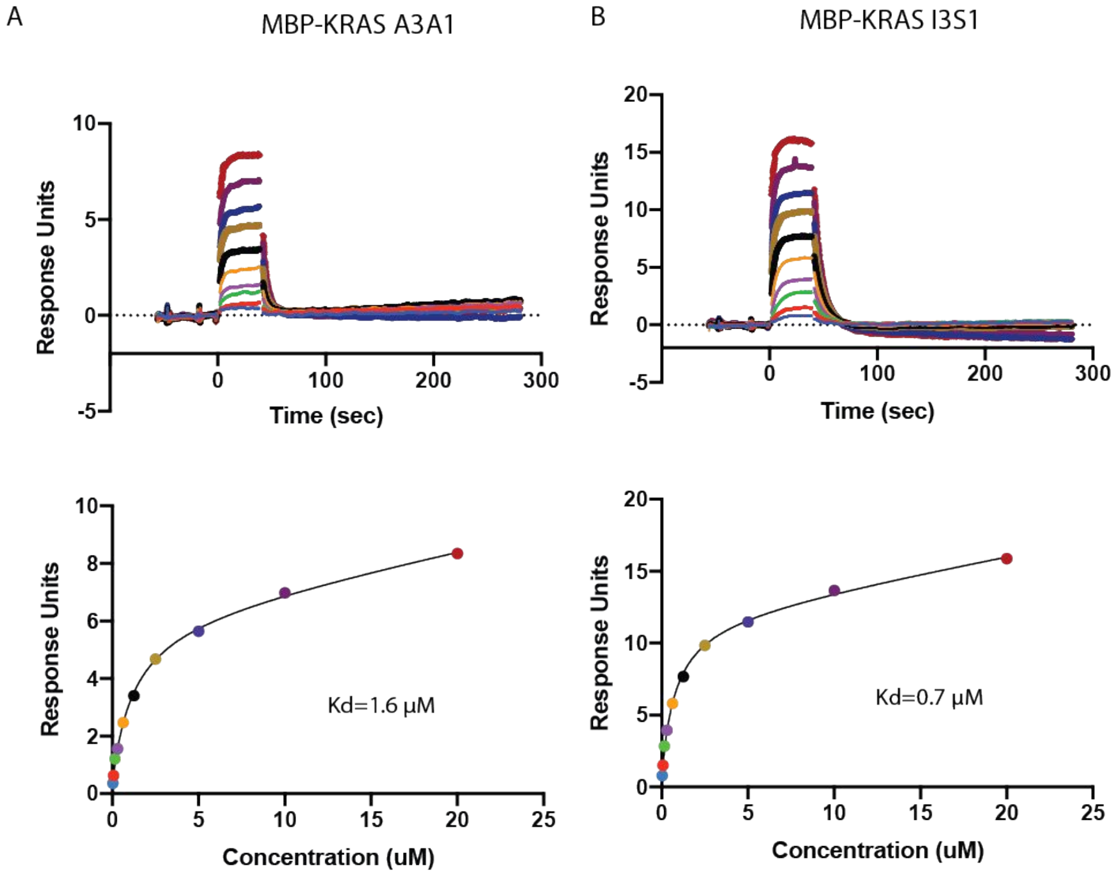

Figure S3. Binding of MBP-KRAS A3A1 and MBP-KRAS I3A1 to PDE6D. A and B. SPR binding curves of PDE6D to MBP-tagged KRAS A3A1 (A) and MBP-tagged KRAS I3S1 (B). PDE6D at concentrations from 0-20 μM. Figures generated using Prism8.

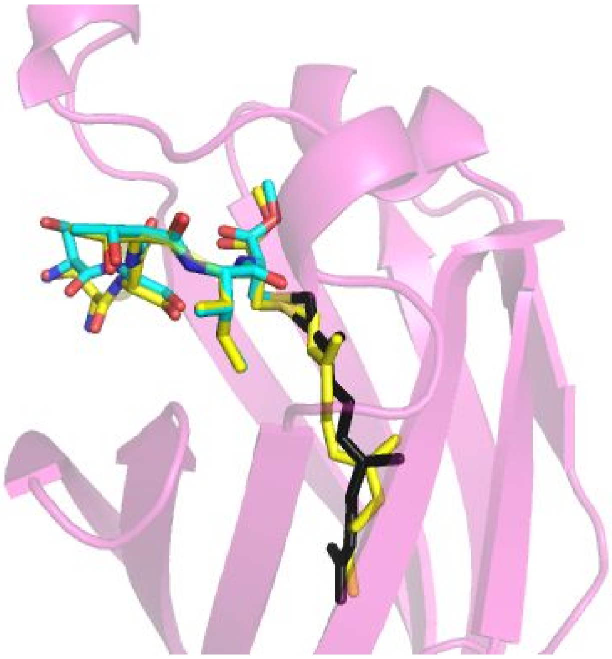

Figure S4. Comparison between KRAS S3I1 and INPP5E conformation when bound to PDE6D. KRAS S3I1 (blue sticks) has the same binding mode as INPP5E (yellow sticks – PDB: 5F2U) when bound to PDE6D (pink cartoon form).

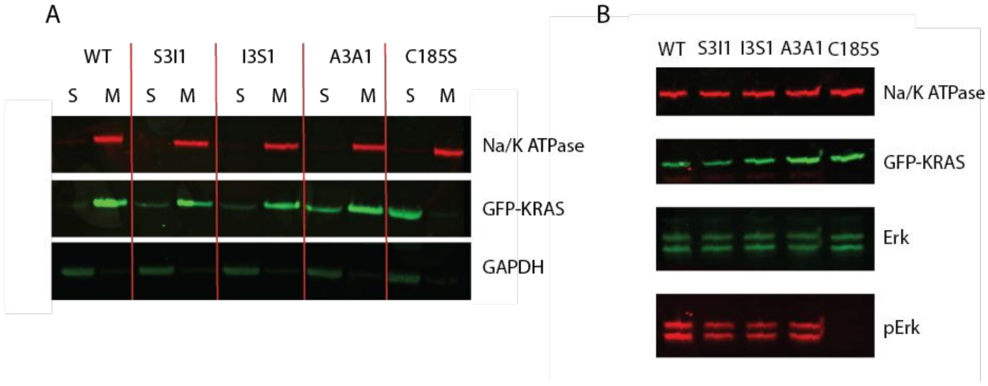

Figure S5. The KRAS S3I1 mutant is membrane binding competent. A. Mutation of the KRAS HVR has minimal effect on membrane binding in cells transfected with GFP-KRAS. B. Single transfection of HEK293F cells results in comparable levels of pErk for all KRAS mutants with the exception of C185S which is not post-translationally modified.

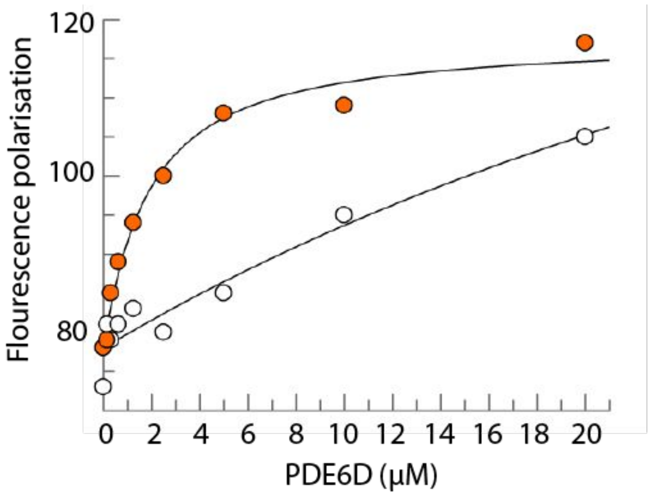

Figure S6. Compound-2 effect on KRAS binding to PDE6D. Shown in red is the binding curve of KRAS at 500 nM to PDE6D. Kd = 1.6 ± 0.6 μM. The measured affinity of KRAS at 500 nM to PDE6D in the presence of 500 μM Compound-2 is 55.2 ± 86.1 μM.

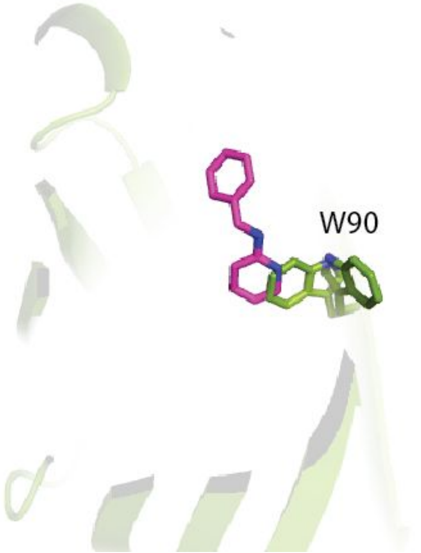

Figure S7. Compound-2 (pink sticks) occupies a site that PDE6D residue W90 (green sticks) can occupy

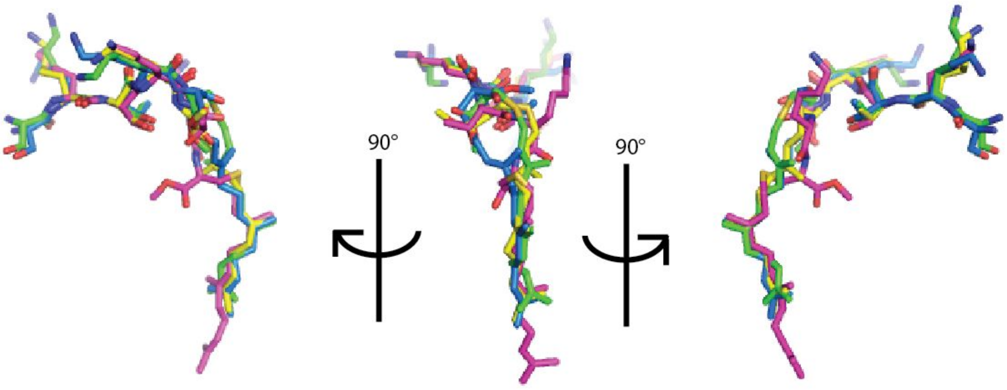

Figure S8. KRAS C-terminal residues have significant conformational heterogeneity when bound to PDE6D. Overlay of KRAS C-terminal residues from PDB: 5TAR (pink), 5TBT(yellow) and both conformations from the KRas:compound-1 complex (blue and pink) show a high level of conformational heterogeneity.

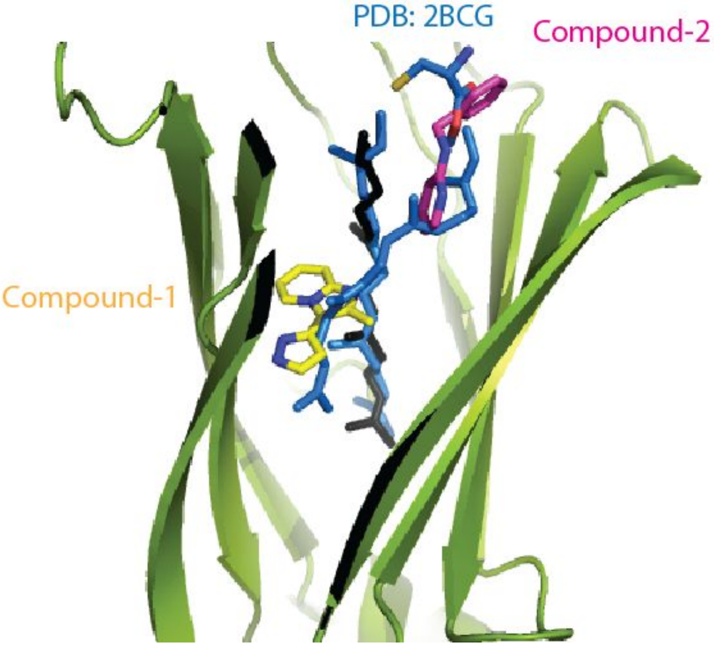

Figure S9. Overlay of double geranylgeranylated cysteine from PDB 2BCG (blue stick form) with our PDE6D: Cys(OMe)-Ger structure (Geranyl shown in black stick form and PDE6D green cartoon form. Position of Compound-1 (yellow sticks) and Compound-2 (pink sticks) at the binding site at the entrance of the PDE6D binding pocket overlay with the second geranylgeranyl group from PDB: 2BCG. 2BCG is the crystal structure of the double geranylgeranylated Ypt1: GDI complex.[1]

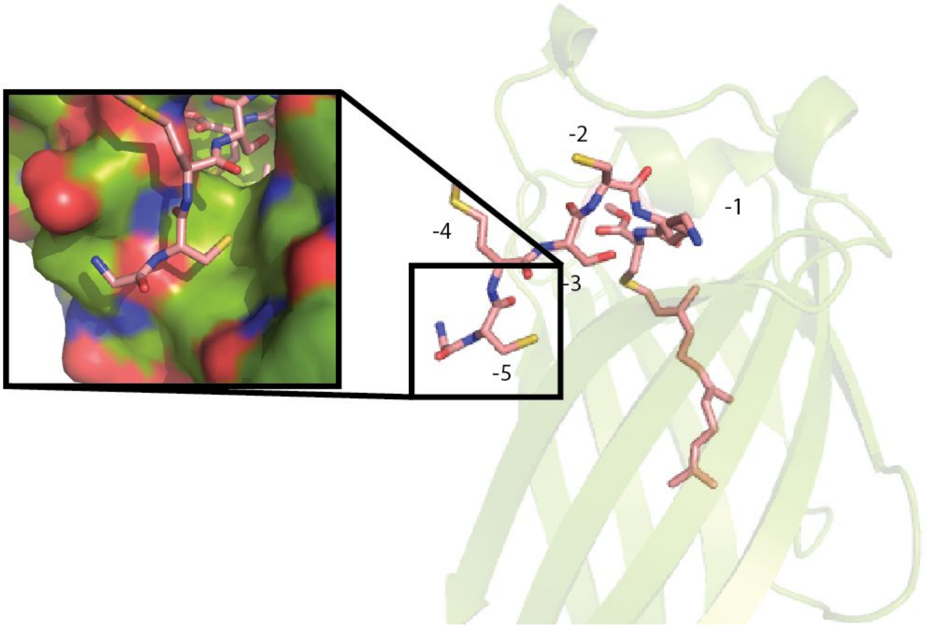

Figure S10. The HRAS cysteine in position -5 is not solvent exposed. Crystal structure of the HRAS: PDE6D complex shows that the cysteine in the -5 position faces PDE6D and is consequently not solvent exposed. HRAS residues are numbers relative to the carboxy-methylated cysteine residue.

A B C

D E

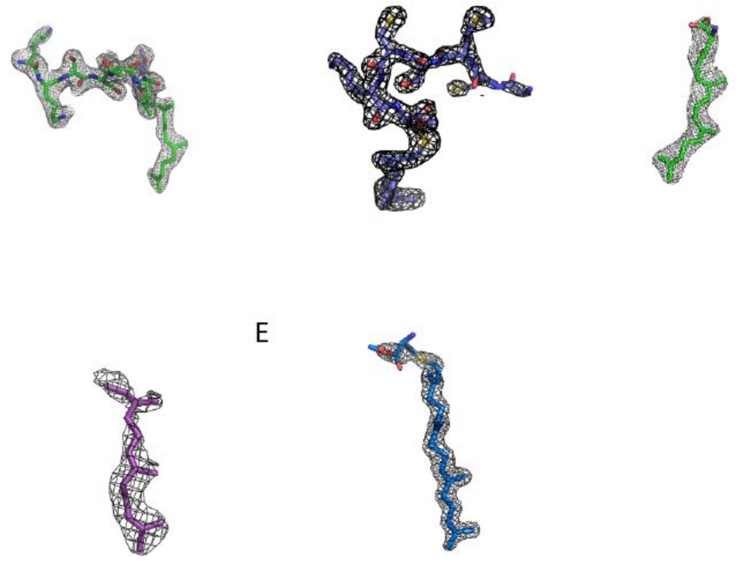

Figure S11. Electron density for bound peptides. (A) representative electron density for PDE6D:KRAS S3I1 complex. (B) representative electron density for HRAS peptide in conformation 1. (C) representative electron density for HRAS in conformation 2. (D) representative electron density for NRAS farnesyl. (E) representative electron density for Cys(Ome)-geranylgeranyl. All at a 1 sigma cut-off.

	PDE6D: KRAS S3I1 complex	PDE6D: HRAS peptide complex	PDE6D: NRAS peptide complex	PDE6D: KRAS peptide: Compound-1	PDE6D: Compound-2 complex	PDE6D: Cys(OMe)-Geranylgeranyl
**PDB code**	7Q9U	7QF9	7Q9R	7Q9S	7QJK	7Q9Q
**Data collection**						
Space group	P 1	P 21 21 21	P 31 2 1	C 2 2 21	P 41 21 2	P1
Cell Dimensions						
a, b, c (Å)	51.00, 57.92, 79.00	34.64, 82.77, 112.45	55.78, 55.78, 114.87	77.08, 81.08, 118.51	76.55, 76.55, 238.02	32.05, 35.29, 37.65
α, β, γ (°)	82.77, 81.72, 68.38	90.00, 90.00, 90.00	90.00, 90.00, 90.00	90.00, 90.00, 90.00	90.00, 90.00, 90.00	77.73, 68.98, 68.23
Resolution (Å)	53.68-2.24	46.52-1.95	48.31-2.50	40.65-1.85	76.55-3.1	34.97-1.45
7777R_{merge}[a]	0.075 (0.294)	0.049 (0.710)	0.046 (0.677)	0.073 (0.735)	0.314(1.919)	3.1 (0.991)
I/σI	6.1 (1.5)	16.9 (1.9)	10.5 (3.2)	15.1 (2.9)	5.6 (1.0)	17.9 (1.8)
Completeness (%)	94.7 (78.9)	99.8 (99.9)	100.0 (100.0)	100.0 (100.0)	90.6 (92.4)	95.9 (93.0)
Redundancy	1.7 (1.4)	6.4 (5.7)	9.5 (9.3)	8.4 (8.7)	8.0 (8.2)	3.5 (3.4)
CC1/2	0.995 (0.742)	0.950 (0.920)	0.995 (0.859)	0.999 (0.880)	0.975 (0.343)	1.000 (0.669)
**Refinement**						
Resolution (Å)	53.68-2.24	46.52-1.95	48.31-2.50	40.65-1.85	76.55-3.1	34.97-1.45
No. of reflections	37,402	23,132	7,256	32,039	12,108	23,214
R_{work}[c]/R_{free}[d]	20.0/24.8	23.2/28.8	23.8/28.0	20.0/22.5	24.7/28.3	23.0/27.6
No. of atoms						
Protein	5,210	2,518	1,222	2,647	4,694	1,235
Ligand	121	50	46	97	79	32
Water	152	137	48	223	26	115
Bfactors						
Protein	50.56	44.62	30.26	32.45	83.59	31.10
Ligand	53.99	55.18	47.23	40.64	78.58	35.91
Water	47.76	48.25	25.48	41.02	60.90	39.81
R.m.s deviation						
Bond lengths (Å)	0.013	0.004	0.013	0.012	0.013	0.13
Bond angles (°)	2.02	0.89	1.52	1.63	1.54	2.06
Ramachandran plot statistics						
Favoured region (%)	97.43	97.64	96.50	98.67	94.50	97.22
Allowed region (%)	2.57	2.03	3.50	1.33	4.08	2.78
Outlier region (%)	0.00	0.34	0.00	0.00	1.42	0.00

Table S1: Crystallographic table of statistics

[a]$R_{merge} = \sum|I_{obs} - I_{avg}|/\sum I_{avg}$

[b]The values for the highest-resolution bin are in parentheses

[c]$R_{work} = \sum|F_{obs} - F_{calc}|/\sum F_{obs}$.

[d]Five percent of the reflection data were selected at random as a test set, and only these data were used to calculate R_{free}

Authors will release the atomic coordinates upon article publication.

List of constructs

His$_6$ – TEV – PDE6D

Plasmid: pET-DUET

DNA sequence:
ATGGGCAGCAGCCATCACCATCATCACCACAGCCAGGATCTGGAAAACCTGTATTTTCAGGGATCCGGA
ATTCTCTCAGCCAAGGACGAGCGGGCCAGGGAGATCCTGAGGGGCTTCAAACTAAATTGGATGAACCTT
CGGGATGCTGAGACAGGGAAGATACTCTGGCAAGGAACAGAAGACCTGTCTGTCCCTGGTGTGGAGCAT
GAAGCCCGTGTTCCCAAGAAAATCCTCAAGTGCAAGGCAGTGTCTCGAGAACTTAATTTTTCTTCGACA
GAACAAATGGAAAAATTCCGCCTGGAACAAAAAGTTTACTTCAAAGGGCAATGCCTAGAAGAATGGTT
CTTCGAGTTTGGCTTTGTGATCCCTAACTCCACAAATACCTGGCAGTCCTTGATAGAGGCAGCACCCGA
GTCCCAGATGATGCCAGCAAGCGTCTTAACTGGGAACGTTATCATAGAAACAAAGTTTTTTGACGACGA
TCTTCTTGTAAGCACATCCAGAGTGAGGCTTTTCTATGTTTAA

Protein sequence:
MGSSHHHHHHSQDLENLYFQGSGILSAKDERAREILRGFKLNWMNLRDAETGKILWQGTEDLSVPGVE
HEARVPKKILKCKAVSRELNFSSTEQMEKFRLEQKVYFKGQCLEEWFFEFGFVIPNSTNTWQSLIEAAPE
SQMMPASVLTGNVIIETKFFDDDLLVSTSRVRLFYV-

Forward primer: TTCCGAATTCTCAGCCAAGGACGAGCG
Reverse primer: TTCCAAGCTTTTAAACATAGAAAAGCCTCAC

His$_8$ –His$_8$ – TEV – PDE6D

Plasmid: pBDDP-SPR3

DNA sequence:
ATGGGCCATCACCATCACCATCACCATCACGGCGCGACCGGCAGCACCGCGGGCAGCGGCACCGCGGGCA
GCACCGGCGCGAGCGGCGCGAGCACCGGCGGCACCGGCGCGACCCATCATCATCATCATCATCATCATGA
AAACCTGTATTTTCAGGGCGGATCCGAATTCTCAGCCAAGGACGAGCGGGCCAGGGAGATCCTGAGGGG
CTTCAAACTAAATTGGATGAACCTTCGGGATGCTGAGACAGGGAAGATACTCTGGCAAGGAACAGAAG
ACCTGTCTGTCCCTGGTGTGGAGCATGAAGCCCGTGTTCCCAAGAAAATCCTCAAGTGCAAGGCAGTGT
CTCGAGAACTTAATTTTTCTTCGACAGAACAAATGGAAAAATTCCGCCTGGAACAAAAAGTTTACTTCA
AAGGGCAATGCCTAGAAGAATGGTTCTTCGAGTTTGGCTTTGTGATCCCTAACTCCACAAATACCTGGC
AGTCCTTGATAGAGGCAGCACCCGAGTCCCAGATGATGCCAGCAAGCGTCTTAACTGGGAACGTTATCA
TAGAAACAAAGTTTTTTGACGACGATCTTCTTGTAAGCACATCCAGAGTGAGGCTTTTCTATGTTTAA

Protein sequence:
MGHHHHHHHHGATGSTAGSGTAGSTGASGASTGGTGATHHHHHHHHENLYFQGGSEFSAKDERAREI
LRGFKLNWMNLRDAETGKILWQGTEDLSVPGVEHEARVPKKILKCKAVSRELNFSSTEQMEKFRLEQK
VYFKGQCLEEWFFEFGFVIPNSTNTWQSLIEAAPESQMMPASVLTGNVIIETKFFDDDLLVSTSRVRLFYV
-

PDE6D-SPR3 For: ATCGGGATCCTCAGCCAAGGCTGAGACAGGG
PDE6D-SPR3 Rev: GACTAAGCTTTTAAACATAGAAAAGCCTCACTCTGG

His$_6$ – TEV PDE6D M20K

Plasmid: pET-DUET

DNA Sequence:
ATGGGCAGCAGCCATCACCATCATCACCACAGCCAGGATCTGGAAAACCTGTATTTTCAGGGATCCGGA
ATTCTCTCAGCCAAGGACGAGCGGGCCAGGGAGATCCTGAGGGGCTTCAAACTAAATTGGAAAAACCTT
CGGGATGCTGAGACAGGGAAGATACTCTGGCAAGGAACAGAAGACCTGTCTGTCCCTGGTGTGGAGCAT
GAAGCCCGTGTTCCCAAGAAAATCCTCAAGTGCAAGGCAGTGTCTCGAGAACTTAATTTTTCTTCGACA
GAACAAATGGAAAAATTCCGCCTGGAACAAAAAGTTTACTTCAAAGGGCAATGCCTAGAAGAATGGTT
CTTCGAGTTTGGCTTTGTGATCCCTAACTCCACAAATACCTGGCAGTCCTTGATAGAGGCAGCACCCGA

GTCCCAGATGATGCCAGCAAGCGTCTTAACTGGGAACGTTATCATAGAAACAAAGTTTTTTGACGACGA
TCTTCTTGTAAGCACATCCAGAGTGAGGCTTTTCTATGTTTAA

Protein Sequence:
MGSSHHHHHHSQDLENLYFQGSGILSAKDERAREILRGFKLNWKNLRDAETGKILWQGTEDLSVPGVE
HEARVPKKILKCKAVSRELNFSSTEQMEKFRLEQKVYFKGQCLEEWFFEFGFVIPNSTNTWQSLIEAAPE
SQMMPASVLTGNVIIETKFFDDDLLVSTSRVRLFYV-

PDE6D M20K Forward: ACTAAATTGGAAAAACCTTCGGGATG
PDE6D M20K Reverse: TTGAAGCCCCTCAGGATC

MKate - PDE6D M20K

Plasmid: pmKate2-C1

DNA sequence:
TCAGCCAAGGACGAGCGGGCCAGGGAGATCCTGAGGGGCTTCAAACTAAATTGGAAAAACCTTCGGGAT
GCTGAGACAGGGAAGATACTCTGGCAAGGAACAGAAGACCTGTCTGTCCCTGGTGTGGAGCATGAAGCC
CGTGTTCCCAAGAAAATCCTCAAGTGCAAGGCAGTGTCTCGAGAACTTAATTTTTCTTCGACAGAACAA
ATGGAAAAATTCCGCCTGGAACAAAAAGTTTACTTCAAAGGGCAATGCCTAGAAGAATGGTTCTTCGA
GTTTGGCTTTGTGATCCCTAACTCCACAAATACCTGGCAGTCCTTGATAGAGGCAGCACCCGAGTCCCA
GATGATGCCAGCAAGCGTCTTAACTGGGAACGTTATCATAGAAACAAAGTTTTTTGACGACGATCTTCT
TGTAAGCACATCCAGAGTGAGGCTTTTCTATGTTTAA

Protein sequence:
SAKDERAREILRGFKLNWKNLRDAETGKILWQGTEDLSVPGVEHEARVPKKILKCKAVSRELNFSSTEQ
MEKFRLEQKVYFKGQCLEEWFFEFGFVIPNSTNTWQSLIEAAPESQMMPASVLTGNVIIETKFFDDDLLV
STSRVRLFYV-

PDE6D M20K Forward: ACTAAATTGGAAAAACCTTCGGGATG
PDE6D M20K Reverse: TTGAAGCCCCTCAGGATC

GFP-KRAS G12D WT

Plasmid: eGFP-C1

DNA sequence:
ATGACAGAATACAAGCTTGTTGTTGTTGGCGCCGACGGTGTGGGCAAGAGTGCGCTGACCATCCAGCTG
ATCCAGAACCATTTTGTGGACGAATACGACCCCACTATAGAGGATTCCTACCGGAAGCAGGTGGTCATT
GATGGGGAGACGTGCCTGTTGGACATCCTGGATACCGCCGGCCAGGAGGAGTACAGCGCCATGCGGGAC
CAGTACATGCGCACCGGGGAGGGCTTCCTGTGTGTGTTTGCCATCAACAACACCAAGTCTTTTGAGGAT
ATCCACCACTACAGGGAGCAGATCAAACGGGTGAAGGACTCGGAAGACGTGCCCATGGTCCTAGTAGGA
AATAAATGTGATTTGCCTTCCAGAACAGTAGACACAAAACAGGCTCAGGACTTAGCAAGAAGTTATGG
AATTCCTTTTATTGAAACATCAGCAAAGACAAGACAGGGTGTTGATGATGCCTTCTATACATTAGTTCG
AGAAATTCGAAAACATAAAGAAAAGATGAGCAAAGATGGTAAAAAGAAGAAAAAGAAGTCAAAGACA
AAGTGTGTAATTATGTAA

Protein sequence:
MTEYKLVVVGAGGVGKSALTIQLIQNHFVDEYDPTIEDSYRKQVVIDGETCLLDILDTAGQEEYSAMRDQ
YMRTGEGFLCVFAINNTKSFEDIHHYREQIKRVKDSEDVPMVLVGNKCDLPSRTVDTKQAQDLARSYGIP
FIETSAKTRQGVDDAFYTLVREIRKHKEKMSKDGKKKKKKSKTKCVIM

GFP-KRas G12D For: TGTTGGCGCCGACGGTGTGGGCA
GFP-KRas G12D Rev:
ACAACAAGCTTGTATTCTGTCATTTACATAATTACACACTTTGTCTTTGACTTCTTTTTCTTCTTTTTA
C

GFP-KRAS G12D S3I1

Plasmid: eGFP-C1

DNA sequence:
ATGACAGAATACAAGCTTGTTGTTGTTGGCGCCGACGGTGTGGGCAAGAGTGCGCTGACCATCCAGCTG
ATCCAGAACCATTTTGTGGACGAATACGACCCCACTATAGAGGATTCCTACCGGAAGCAGGTGGTCATT
GATGGGGAGACGTGCCTGTTGGACATCCTGGATACCGCCGGCCAGGAGGAGTACAGCGCCATGCGGGAC
CAGTACATGCGCACCGGGGAGGGCTTCCTGTGTGTGTTTGCCATCAACAACACCAAGTCTTTTGAGGAT
ATCCACCACTACAGGGAGCAGATCAAACGGGTGAAGGACTCGGAAGACGTGCCCATGGTCCTAGTAGGA
AATAAATGTGATTTGCCTTCCAGAACAGTAGACACAAAACAGGCTCAGGACTTAGCAAGAAGTTATGG
AATTCCTTTTATTGAAACATCAGCAAAGACAAGACAGGGTGTTGATGATGCCTTCTATACATTAGTTCG
AGAAATTCGAAAACATAAAGAAAAGATGAGCAAAGATGGTAAAAAGAAGAAAAAGAAGTCATCCACA
ATCTGTGTAATTATGTAA

Protein sequence:
MTEYKLVVVGADGVGKSALTIQLIQNHFVDEYDPTIEDSYRKQVVIDGETCLLDILDTAGQEEYSAMRDQ
YMRTGEGFLCVFAINNTKSFEDIHHYREQIKRVKDSEDVPMVLVGNKCDLPSRTVDTKQAQDLARSYGIP
FIETSAKTRQGVDDAFYTLVREIRKHKEKMSKDGKKKKKKSSTICVIM-

GFP-KRas S3I1 Forward: AATCTGTGTAATTATGTAAATGACTG
GFP-KRas S3I1 Reverse: GTGGATGACTTCTTTTTCTTCTTTTTAC

GFP-KRAS G12D I3S1

Plasmid: eGFP-C1

DNA Sequence:
ATGACAGAATACAAGCTTGTTGTTGTTGGCGCCGACGGTGTGGGCAAGAGTGCGCTGACCATCCAGCTG
ATCCAGAACCATTTTGTGGACGAATACGACCCCACTATAGAGGATTCCTACCGGAAGCAGGTGGTCATT
GATGGGGAGACGTGCCTGTTGGACATCCTGGATACCGCCGGCCAGGAGGAGTACAGCGCCATGCGGGAC
CAGTACATGCGCACCGGGGAGGGCTTCCTGTGTGTGTTTGCCATCAACAACACCAAGTCTTTTGAGGAT
ATCCACCACTACAGGGAGCAGATCAAACGGGTGAAGGACTCGGAAGACGTGCCCATGGTCCTAGTAGGA
AATAAATGTGATTTGCCTTCCAGAACAGTAGACACAAAACAGGCTCAGGACTTAGCAAGAAGTTATGG
AATTCCTTTTATTGAAACATCAGCAAAGACAAGACAGGGTGTTGATGATGCCTTCTATACATTAGTTCG
AGAAATTCGAAAACATAAAGAAAAGATGAGCAAAGATGGTAAAAAGAAGAAAAAGAAGTCAATCACA
TCCTGTGTAATTATGTAA

Protein sequence:
MTEYKLVVVGADGVGKSALTIQLIQNHFVDEYDPTIEDSYRKQVVIDGETCLLDILDTAGQEEYSAMRDQ
YMRTGEGFLCVFAINNTKSFEDIHHYREQIKRVKDSEDVPMVLVGNKCDLPSRTVDTKQAQDLARSYGIP
FIETSAKTRQGVDDAFYTLVREIRKHKEKMSKDGKKKKKKSITSCVIM-

GFP-KRas S3I1 Forward: ATCCTGTGTAATTATGTAAATGACAG
GFP-KRas S3I1 Reverse: GTGATTGACTTCTTTTTCTTCTTTTTAC

GFP-KRAS G12D A3A1

Plasmid: eGFP-C1

DNA Sequence:
ATGACAGAATACAAGCTTGTTGTTGTTGGCGCCGACGGTGTGGGCAAGAGTGCGCTGACCATCCAGCTG
ATCCAGAACCATTTTGTGGACGAATACGACCCCACTATAGAGGATTCCTACCGGAAGCAGGTGGTCATT
GATGGGGAGACGTGCCTGTTGGACATCCTGGATACCGCCGGCCAGGAGGAGTACAGCGCCATGCGGGAC
CAGTACATGCGCACCGGGGAGGGCTTCCTGTGTGTGTTTGCCATCAACAACACCAAGTCTTTTGAGGAT
ATCCACCACTACAGGGAGCAGATCAAACGGGTGAAGGACTCGGAAGACGTGCCCATGGTCCTAGTAGGA
AATAAATGTGATTTGCCTTCCAGAACAGTAGACACAAAACAGGCTCAGGACTTAGCAAGAAGTTATGG
AATTCCTTTTATTGAAACATCAGCAAAGACAAGACAGGGTGTTGATGATGCCTTCTATACATTAGTTCG
AGAAATTCGAAAACATAAAGAAAAGATGAGCAAAGATGGTAAAAAGAAGAAAAAGAAGTCAGCCACA
GCCTGTGTAATTATGTAA

Protein sequence:

MTEYKLVVVGADGVGKSALTIQLIQNHFVDEYDPTIEDSYRKQVVIDGETCLLDILDTAGQEEYSAMRDQ
YMRTGEGFLCVFAINNTKSFEDIHHYREQIKRVKDSEDVPMVLVGNKCDLPSRTVDTKQAQDLARSYGIP
FIETSAKTRQGVDDAFYTLVREIRKHKEKMSKDGKKKKKKSATACVIM-

GFP-KRas A3A1 Forward: AGCCTGTGTAATTATGTAAATGACTG
GFP-KRas A3A1 Reverse: GTGGCTGACTTCTTTTTCTTCTTTTTAC

GFP-KRAS G12D A3A1

Plasmid: eGFP-C1

DNA Sequence:
ATGACAGAATACAAGCTTGTTGTTGTTGGCGCCGACGGTGTGGGCAAGAGTGCGCTGACCATCCAGCTG
ATCCAGAACCATTTTGTGGACGAATACGACCCCACTATAGAGGATTCCTACCGGAAGCAGGTGGTCATT
GATGGGGAGACGTGCCTGTTGGACATCCTGGATACCGCCGGCCAGGAGGAGTACAGCGCCATGCGGGAC
CAGTACATGCGCACCGGGGAGGGCTTCCTGTGTGTGTTTGCCATCAACAACACCAAGTCTTTTGAGGAT
ATCCACCACTACAGGGAGCAGATCAAACGGGTGAAGGACTCGGAAGACGTGCCCATGGTCCTAGTAGGA
AATAAATGTGATTTGCCTTCCAGAACAGTAGACACAAAACAGGCTCAGGACTTAGCAAGAAGTTATGG
AATTCCTTTTATTGAAACATCAGCAAAGACAAGACAGGGTGTTGATGATGCCTTCTATACATTAGTTCG
AGAAATTCGAAAACATAAAGAAAAGATGAGCAAAGATGGTAAAAAGAAGAAAAAGAAGTCAAAGACA
AAGGCTGTAATTATGTAA

Protein sequence:
MTEYKLVVVGADGVGKSALTIQLIQNHFVDEYDPTIEDSYRKQVVIDGETCLLDILDTAGQEEYSAMRDQ
YMRTGEGFLCVFAINNTKSFEDIHHYREQIKRVKDSEDVPMVLVGNKCDLPSRTVDTKQAQDLARSYGIP
FIETSAKTRQGVDDAFYTLVREIRKHKEKMSKDGKKKKKKSKTKAVIM-

GFP-KRas C185A Forward: AAAGACAAAGGCTGTAATTATGTAAATGACTGAATATAAAC
GFP-KRas C185A Reverse: GACTTCTTTTTCTTCTTTTTACC

untagged-KRAS G12D I3S1

Plasmid: pcDNA3.1

DNA Sequence:
ATGACAGAATACAAGCTTGTTGTTGTTGGCGCCGGCGGTGTGGGCAAGAGTGCGCTGACCATCCAGCTG
ATCCAGAACCATTTTGTGGACGAATACGACCCCACTATAGAGGATTCCTACCGGAAGCAGGTGGTCATT
GATGGGGAGACGTGCCTGTTGGACATCCTGGATACCGCCGGCCAGGAGGAGTACAGCGCCATGCGGGAC
CAGTACATGCGCACCGGGGAGGGCTTCCTGTGTGTGTTTGCCATCAACAACACCAAGTCTTTTGAGGAT
ATCCACCACTACAGGGAGCAGATCAAACGGGTGAAGGACTCGGAAGACGTGCCCATGGTCCTAGTAGGA
AATAAATGTGATTTGCCTTCCAGAACAGTAGACACAAAACAGGCTCAGGACTTAGCAAGAAGTTATGG
AATTCCTTTTATTGAAACATCAGCAAAGACAAGACAGGGTGTTGATGATGCCTTCTATACATTAGTTCG
AGAAATTCGAAAACATAAAGAAAAGATGAGCAAAGATGGTAAAAAGAAGAAAAAGAAGTCAATCACA
TCCTGTGTAATTATGTAA

Protein sequence:
MTEYKLVVVGADGVGKSALTIQLIQNHFVDEYDPTIEDSYRKQVVIDGETCLLDILDTAGQEEYSAMRDQ
YMRTGEGFLCVFAINNTKSFEDIHHYREQIKRVKDSEDVPMVLVGNKCDLPSRTVDTKQAQDLARSYGIP
FIETSAKTRQGVDDAFYTLVREIRKHKEKMSKDGKKKKKKSITSCVIM-

Untagged-KRas S3I1 Forward: GATCGGTACCGCCACCATGACAGAATACAAGCTTGTTG
Untagged-KRas S3I1 Reverse: GATCGGATCCTTATTATTACATAATTACACAGATTGTGGATG

His-MBP-tagged KRAS WT

Plasmid: pcDNA3.1

DNA Sequence:

CGGGGTACCGCCACCATGTCACACCATCACCATCATCACCACCATCATCATCACCATGGGTCAGGTTCAG
GATCCGGTATGAAGATCGAGGAGGGCAAGCTTGTGATTTGGATCAACGGCGACAAGGGGTACAACGGC
CTGGCCGAAGTTGGTAAAAAATTCGAAAAAGACACGGGGATCAAAGTCACAGTAGAGCATCCGGACAA
GCTCGAGGAAAAATTTCCTCAGGTGGCAGCCACCGGTGACGGGCCTGACATCATCTTTTGGGCACACGA
TCGGTTCGGCGGCTACGCGCAAAGCGGACTTCTTGCTGAGATCACCCCCGATAAGGCTTTCCAAGACAA
GCTTTATCCGTTTACATGGGATGCAGTTCGATATAACGGTAAACTCATCGCTTATCCCATAGCGGTGGA
GGCCCTGAGTCTGATCTACAACAAAGACCTGCTTCCTAATCCCCCCAAGACATGGGAGGAGATTCCAGC
GCTCGATAAGGAGCTTAAAGCAAAGGGAAAAAGCGCATTGATGTTTAATTTGCAAGAGCCATATTTTA
CCTGGCCCCTGATTGCAGCAGATGGTGGCTATGCCTTTAAGTATGAAAACGGAAAGTATGACATAAAGG
ACGTTGGGGTTGATAATGCAGGAGCCAAGGCTGGATTGACCTTTCTGGTAGATCTTATTAAGAACAAG
CATATGAACGCGGACACGGACTACTCTATCGCTGAAGCAGCGTTTAATAAAGGAGAAACAGCCATGACC
ATAAATGGACCCTGGGCATGGAGCAACATCGACACGTCTAAAGTAAACTACGGTGTTACGGTGCTCCCC
ACCTTCAAGGGCCAACCTTCCAAACCTTTTGTGGGCGTACTGTCAGCAGGGATAAACGCCGCATCCCCC
AACAAGGAGCTGGCTAAAGAGTTCCTTGAAAATTACCTTCTCACTGATGAAGGTCTCGAGGCGGTGAAC
AAAGACAAACCTCTTGGCGCCGTGGCACTTAAGTCATATGAAGAGGAACTGGTAAAAGACCCTAGGATC
GCAGCCACCATGGAGAACGCTCAGAAGGGCGAAATCATGCCTAACATTCCCCAAATGAGCGCCTTTTGG
TATGCAGTTAGAACCGCCGTGATAAATGCAGCCTCCGGCCGCCAAACTGTAGACGAAGCCCTCAAGGAT
GCTCAGACGGGATCAGGGAGTGGCAGTGGTTCCGAAAATCTTTACTTCCAGGGTTCCGGTTCAGGTTCC
ACCGAATACAAGTTGGTAGTCGTTGGAGCTGGCGGCGTTGGAAAATCTGCTCTGACAATCCAGCTTATC
CAAAATCATTTCGTTGACGAGTACGATCCTACCATAGAAGACTCTTACAGAAAACAGGTGGTGATTGAT
GGGGAAACCTGCCTGCTTGACATACTTGACACAGCTGGCCAAGAAGAATATTCCGCCATGCGAGACCAG
TACATGCGCACTGGCGAAGGCTTTCTGTGTGTCTTCGCTATTAATAACACTAAGTCATTCGAAGACATA
CACCATTACCGCGAACAAATAAAACGCGTCAAGGATAGCGAGGACGTGCCCATGGTTCTCGTAGGTAAT
AAGTGCGATCTGCCAAGCAGAACCGTAGACACTAAACAGGCGCAAGATCTTGCCCGGTCTTATGGCATT
CCATTTATTGAGACTTCTGCCAAAACGAGGCAAGGCGTTGATGATGCTTTTTATACACTCGTACGGGAA
ATAAGGAAGCACAAAGAGAAGATGAGTAAGGATGGCAAGAAGAAAAAAAAGAAATCCAAAACCAAGT
GTGTCATAATGTGATAATAAGCGGCCGCAAAAGGAAAA

Protein sequence:
MSHHHHHHHHHHHHGSGSGSGMKIEEGKLVIWINGDKGYNGLAEVGKKFEKDTGIKVTVEHPDKLEEK
FPQVAATGDGPDIIFWAHDRFGGYAQSGLLAEITPDKAFQDKLYPFTWDAVRYNGKLIAYPIAVEALSLIY
NKDLLPNPPKTWEEIPALDKELKAKGKSALMFNLQEPYFTWPLIAADGGYAFKYENGKYDIKDVGVDN
AGAKAGLTFLVDLIKNKHMNADTDYSIAEAAFNKGETAMTINGPWAWSNIDTSKVNYGVTVLPTFKGQ
PSKPFVGVLSAGINAASPNKELAKEFLENYLLTDEGLEAVNKDKPLGAVALKSYEEELVKDPRIAATMEN
AQKGEIMPNIPQMSAFWYAVRTAVINAASGRQTVDEALKDAQTGSGSGSGSENLYFQGSGSGSTEYKLVV
VGAGGVGKSALTIQLIQNHFVDEYDPTIEDSYRKQVVIDGETCLLDILDTAGQEEYSAMRDQYMRTGEGF
LCVFAINNTKSFEDIHHYREQIKRVKDSEDVPMVLVGNKCDLPSRTVDTKQAQDLARSYGIPFIETSAKTR
QGVDDAFYTLVREIRKHKEKMSKDGKKKKKKSKTKCVIM-

His-MBP-tagged KRAS S3I1

Plasmid: pcDNA3.1

DNA Sequence:
CGGGGTACCGCCACCATGTCACACCATCACCATCATCACCACCATCATCATCACCATGGGTCAGGTTCAG
GATCCGGTATGAAGATCGAGGAGGGCAAGCTTGTGATTTGGATCAACGGCGACAAGGGGTACAACGGC
CTGGCCGAAGTTGGTAAAAAATTCGAAAAAGACACGGGGATCAAAGTCACAGTAGAGCATCCGGACAA
GCTCGAGGAAAAATTTCCTCAGGTGGCAGCCACCGGTGACGGGCCTGACATCATCTTTTGGGCACACGA
TCGGTTCGGCGGCTACGCGCAAAGCGGACTTCTTGCTGAGATCACCCCCGATAAGGCTTTCCAAGACAA
GCTTTATCCGTTTACATGGGATGCAGTTCGATATAACGGTAAACTCATCGCTTATCCCATAGCGGTGGA
GGCCCTGAGTCTGATCTACAACAAAGACCTGCTTCCTAATCCCCCCAAGACATGGGAGGAGATTCCAGC
GCTCGATAAGGAGCTTAAAGCAAAGGGAAAAAGCGCATTGATGTTTAATTTGCAAGAGCCATATTTTA
CCTGGCCCCTGATTGCAGCAGATGGTGGCTATGCCTTTAAGTATGAAAACGGAAAGTATGACATAAAGG
ACGTTGGGGTTGATAATGCAGGAGCCAAGGCTGGATTGACCTTTCTGGTAGATCTTATTAAGAACAAG
CATATGAACGCGGACACGGACTACTCTATCGCTGAAGCAGCGTTTAATAAAGGAGAAACAGCCATGACC
ATAAATGGACCCTGGGCATGGAGCAACATCGACACGTCTAAAGTAAACTACGGTGTTACGGTGCTCCCC
ACCTTCAAGGGCCAACCTTCCAAACCTTTTGTGGGCGTACTGTCAGCAGGGATAAACGCCGCATCCCCC
AACAAGGAGCTGGCTAAAGAGTTCCTTGAAAATTACCTTCTCACTGATGAAGGTCTCGAGGCGGTGAAC
AAAGACAAACCTCTTGGCGCCGTGGCACTTAAGTCATATGAAGAGGAACTGGTAAAAGACCCTAGGATC

GCAGCCACCATGGAGAACGCTCAGAAGGGCGAAATCATGCCTAACATTCCCCAAATGAGCGCCTTTTGG
TATGCAGTTAGAACCGCCGTGATAAATGCAGCCTCCGGCCGCCAAACTGTAGACGAAGCCCTCAAGGAT
GCTCAGACGGGATCAGGGAGTGGCAGTGGTTCCGAAAATCTTTACTTCCAGGGTTCCGGTTCAGGTTCC
ACCGAATACAAGTTGGTAGTCGTTGGAGCTGGCGGCGTTGGAAAATCTGCTCTGACAATCCAGCTTATC
CAAAATCATTTCGTTGACGAGTACGATCCTACCATAGAAGACTCTTACAGAAAACAGGTGGTGATTGAT
GGGGAAACCTGCCTGCTTGACATACTTGACACAGCTGGCCAAGAAGAATATTCCGCCATGCGAGACCAG
TACATGCGCACTGGCGAAGGCTTTCTGTGTGTCTTCGCTATTAATAACACTAAGTCATTCGAAGACATA
CACCATTACCGCGAACAAATAAAACGCGTCAAGGATAGCGAGGACGTGCCCATGGTTCTCGTAGGTAAT
AAGTGCGATCTGCCAAGCAGAACCGTAGACACTAAACAGGCGCAAGATCTTGCCCGGTCTTATGGCATT
CCATTTATTGAGACTTCTGCCAAAACGAGGCAAGGCGTTGATGATGCTTTTTATACACTCGTACGGGAA
ATAAGGAAGCACAAAGAGAAGATGAGTAAGGATGGCAAGAAGAAAAAAAAGAAATCCAGCACCATCTG
TGTCATAATGTGATAATAAGCGGCCGCAAAAGGAAAA

Protein sequence:
MSHHHHHHHHHHHHGSGSGSGMKIEEGKLVIWINGDKGYNGLAEVGKKFEKDTGIKVTVEHPDKLEEK
FPQVAATGDGPDIIFWAHDRFGGYAQSGLLAEITPDKAFQDKLYPFTWDAVRYNGKLIAYPIAVEALSLIY
NKDLLPNPPKTWEEIPALDKELKAKGKSALMFNLQEPYFTWPLIAADGGYAFKYENGKYDIKDVGVDN
AGAKAGLTFLVDLIKNKHMNADTDYSIAEAAFNKGETAMTINGPWAWSNIDTSKVNYGVTVLPTFKGQ
PSKPFVGVLSAGINAASPNKELAKEFLENYLLTDEGLEAVNKDKPLGAVALKSYEEELVKDPRIAATMEN
AQKGEIMPNIPQMSAFWYAVRTAVINAASGRQTVDEALKDAQTGSGSGSGSENLYFQGSGSGSTEYKLVV
VGAGGVGKSALTIQLIQNHFVDEYDPTIEDSYRKQVVIDGETCLLDILDTAGQEEYSAMRDQYMRTGEGF
LCVFAINNTKSFEDIHHYREQIKRVKDSEDVPMVLVGNKCDLPSRTVDTKQAQDLARSYGIPFIETSAKTR
QGVDDAFYTLVREIRKHKEKMSKDGKKKKKKSSTICVIM-

MBP-KRas S3I1 Forward: CATCTGTGTCATAATGTGATAATAAG
MBP-KRas S3I1 Reverse: GTGCTtGGATTTCTTTTTTTTCTTCTTGC

His-MBP-tagged KRAS I3S1

Plasmid: pcDNA3.1

DNA Sequence:
CGGGGTACCGCCACCATGTCACACCATCACCATCATCACCACCATCATCATCACCATGGGTCAGGTTCAG
GATCCGGTATGAAGATCGAGGAGGGCAAGCTTGTGATTTGGATCAACGGCGACAAGGGGTACAACGGC
CTGGCCGAAGTTGGTAAAAAATTCGAAAAAGACACGGGGATCAAAGTCACAGTAGAGCATCCGGACAA
GCTCGAGGAAAAATTTCCTCAGGTGGCAGCCACCGGTGACGGGCCTGACATCATCTTTTGGGCACACGA
TCGGTTCGGCGGCTACGCGCAAAGCGGACTTCTTGCTGAGATCACCCCCGATAAGGCTTTCCAAGACAA
GCTTTATCCGTTTACATGGGATGCAGTTCGATATAACGGTAAACTCATCGCTTATCCCATAGCGGTGGA
GGCCCTGAGTCTGATCTACAACAAAGACCTGCTTCCTAATCCCCCCAAGACATGGGAGGAGATTCCAGC
GCTCGATAAGGAGCTTAAAGCAAAGGGAAAAAGCGCATTGATGTTTAATTTGCAAGAGCCATATTTTA
CCTGGCCCCTGATTGCAGCAGATGGTGGCTATGCCTTTAAGTATGAAAACGGAAAGTATGACATAAAGG
ACGTTGGGGTTGATAATGCAGGAGCCAAGGCTGGATTGACCTTTCTGGTAGATCTTATTAAGAACAAG
CATATGAACGCGGACACGGACTACTCTATCGCTGAAGCAGCGTTTAATAAAGGAGAAACAGCCATGACC
ATAAATGGACCCTGGGCATGGAGCAACATCGACACGTCTAAAGTAAACTACGGTGTTACGGTGCTCCCC
ACCTTCAAGGGCCAACCTTCCAAACCTTTTGTGGGCGTACTGTCAGCAGGGATAAACGCCGCATCCCCC
AACAAGGAGCTGGCTAAAGAGTTCCTTGAAAATTACCTTCTCACTGATGAAGGTCTCGAGGCGGTGAAC
AAAGACAAACCTCTTGGCGCCGTGGCACTTAAGTCATATGAAGAGGAACTGGTAAAAGACCCTAGGATC
GCAGCCACCATGGAGAACGCTCAGAAGGGCGAAATCATGCCTAACATTCCCCAAATGAGCGCCTTTTGG
TATGCAGTTAGAACCGCCGTGATAAATGCAGCCTCCGGCCGCCAAACTGTAGACGAAGCCCTCAAGGAT
GCTCAGACGGGATCAGGGAGTGGCAGTGGTTCCGAAAATCTTTACTTCCAGGGTTCCGGTTCAGGTTCC
ACCGAATACAAGTTGGTAGTCGTTGGAGCTGGCGGCGTTGGAAAATCTGCTCTGACAATCCAGCTTATC
CAAAATCATTTCGTTGACGAGTACGATCCTACCATAGAAGACTCTTACAGAAAACAGGTGGTGATTGAT
GGGGAAACCTGCCTGCTTGACATACTTGACACAGCTGGCCAAGAAGAATATTCCGCCATGCGAGACCAG
TACATGCGCACTGGCGAAGGCTTTCTGTGTGTCTTCGCTATTAATAACACTAAGTCATTCGAAGACATA
CACCATTACCGCGAACAAATAAAACGCGTCAAGGATAGCGAGGACGTGCCCATGGTTCTCGTAGGTAAT
AAGTGCGATCTGCCAAGCAGAACCGTAGACACTAAACAGGCGCAAGATCTTGCCCGGTCTTATGGCATT
CCATTTATTGAGACTTCTGCCAAAACGAGGCAAGGCGTTGATGATGCTTTTTATACACTCGTACGGGAA
ATAAGGAAGCACAAAGAGAAGATGAGTAAGGATGGCAAGAAGAAAAAAAAGAAATCCATCACCTCCTG
TGTCATAATGTGATAATAAGCGGCCGCAAAAGGAAAA

Protein sequence:
MSHHHHHHHHHHHHGSGSGSGMKIEEGKLVIWINGDKGYNGLAEVGKKFEKDTGIKVTVEHPDKLEEK
FPQVAATGDGPDIIFWAHDRFGGYAQSGLLAEITPDKAFQDKLYPFTWDAVRYNGKLIAYPIAVEALSLIY
NKDLLPNPPKTWEEIPALDKELKAKGKSALMFNLQEPYFTWPLIAADGGYAFKYENGKYDIKDVGVDN
AGAKAGLTFLVDLIKNKHMNADTDYSIAEAAFNKGETAMTINGPWAWSNIDTSKVNYGVTVLPTFKGQ
PSKPFVGVLSAGINAASPNKELAKEFLENYLLTDEGLEAVNKDKPLGAVALKSYEEELVKDPRIAATMEN
AQKGEIMPNIPQMSAFWYAVRTAVINAASGRQTVDEALKDAQTGSGSGSGSENLYFQGSGSGSTEYKLVV
VGAGGVGKSALTIQLIQNHFVDEYDPTIEDSYRKQVVIDGETCLLDILDTAGQEEYSAMRDQYMRTGEGF
LCVFAINNTKSFEDIHHYREQIKRVKDSEDVPMVLVGNKCDLPSRTVDTKQAQDLARSYGIPFIETSAKTR
QGVDDAFYTLVREIRKHKEKMSKDGKKKKKKSITSCVIM-

MBP-KRas S3I1 Forward: CTCCTGTGTCATAATGTGATAATAAG
MBP-KRas S3I1 Reverse: GTGATGGATTTCTTTTTTTCTTCTTGC

His-MBP-tagged KRAS WT

Plasmid: pcDNA3.1

DNA Sequence:
CGGGGTACCGCCACCATGTCACACCATCACCATCATCACCACCATCATCATCACCATGGGTCAGGTTCAG
GATCCGGTATGAAGATCGAGGAGGGCAAGCTTGTGATTTGGATCAACGGCGACAAGGGGTACAACGGC
CTGGCCGAAGTTGGTAAAAAATTCGAAAAAGACACGGGGATCAAAGTCACAGTAGAGCATCCGGACAA
GCTCGAGGAAAAATTTCCTCAGGTGGCAGCCACCGGTGACGGGCCTGACATCATCTTTTGGGCACACGA
TCGGTTCGGCGGCTACGCGCAAAGCGGACTTCTTGCTGAGATCACCCCCGATAAGGCTTTCCAAGACAA
GCTTTATCCGTTTACATGGGATGCAGTTCGATATAACGGTAAACTCATCGCTTATCCCATAGCGGTGGA
GGCCCTGAGTCTGATCTACAACAAAGACCTGCTTCCTAATCCCCCCAAGACATGGGAGGAGATTCCAGC
GCTCGATAAGGAGCTTAAAGCAAAGGGAAAAAGCGCATTGATGTTTAATTTGCAAGAGCCATATTTTA
CCTGGCCCCTGATTGCAGCAGATGGTGGCTATGCCTTTAAGTATGAAAACGGAAAGTATGACATAAAGG
ACGTTGGGGTTGATAATGCAGGAGCCAAGGCTGGATTGACCTTTCTGGTAGATCTTATTAAGAACAAG
CATATGAACGCGGACACGGACTACTCTATCGCTGAAGCAGCGTTTAATAAAGGAGAAACAGCCATGACC
ATAAATGGACCCTGGGCATGGAGCAACATCGACACGTCTAAAGTAAACTACGGTGTTACGGTGCTCCCC
ACCTTCAAGGGCCAACCTTCCAAACCTTTTGTGGGCGTACTGTCAGCAGGGATAAACGCCGCATCCCCC
AACAAGGAGCTGGCTAAAGAGTTCCTTGAAAATTACCTTCTCACTGATGAAGGTCTCGAGGCGGTGAAC
AAAGACAAACCTCTTGGCGCCGTGGCACTTAAGTCATATGAAGAGGAACTGGTAAAAGACCCTAGGATC
GCAGCCACCATGGAGAACGCTCAGAAGGGCGAAATCATGCCTAACATTCCCCAAATGAGCGCCTTTTGG
TATGCAGTTAGAACCGCCGTGATAAATGCAGCCTCCGGCCGCCAAACTGTAGACGAAGCCCTCAAGGAT
GCTCAGACGGGATCAGGGAGTGGCAGTGGTTCCGAAAATCTTTACTTCCAGGGTTCCGGTTCAGGTTCC
ACCGAATACAAGTTGGTAGTCGTTGGAGCTGGCGGCGTTGGAAAATCTGCTCTGACAATCCAGCTTATC
CAAAATCATTTCGTTGACGAGTACGATCCTACCATAGAAGACTCTTACAGAAAACAGGTGGTGATTGAT
GGGGAAACCTGCCTGCTTGACATACTTGACACAGCTGGCCAAGAAGAATATTCCGCCATGCGAGACCAG
TACATGCGCACTGGCGAAGGCTTTCTGTGTGTCTTCGCTATTAATAACACTAAGTCATTCGAAGACATA
CACCATTACCGCGAACAAATAAAACGCGTCAAGGATAGCGAGGACGTGCCCATGGTTCTCGTAGGTAAT
AAGTGCGATCTGCCAAGCAGAACCGTAGACACTAAACAGGCGCAAGATCTTGCCCGGTCTTATGGCATT
CCATTTATTGAGACTTCTGCCAAAACGAGGCAAGGCGTTGATGATGCTTTTTATACACTCGTACGGGAA
ATAAGGAAGCACAAAGAGAAGATGAGTAAGGATGGCAAGAAGAAAAAAAAGAAATCCGCCACCGCCTG
TGTCATAATGTGATAATAAGCGGCCGCAAAAGGAAAA

Protein sequence:
MSHHHHHHHHHHHHGSGSGSGMKIEEGKLVIWINGDKGYNGLAEVGKKFEKDTGIKVTVEHPDKLEEK
FPQVAATGDGPDIIFWAHDRFGGYAQSGLLAEITPDKAFQDKLYPFTWDAVRYNGKLIAYPIAVEALSLIY
NKDLLPNPPKTWEEIPALDKELKAKGKSALMFNLQEPYFTWPLIAADGGYAFKYENGKYDIKDVGVDN
AGAKAGLTFLVDLIKNKHMNADTDYSIAEAAFNKGETAMTINGPWAWSNIDTSKVNYGVTVLPTFKGQ
PSKPFVGVLSAGINAASPNKELAKEFLENYLLTDEGLEAVNKDKPLGAVALKSYEEELVKDPRIAATMEN
AQKGEIMPNIPQMSAFWYAVRTAVINAASGRQTVDEALKDAQTGSGSGSGSENLYFQGSGSGSTEYKLVV
VGAGGVGKSALTIQLIQNHFVDEYDPTIEDSYRKQVVIDGETCLLDILDTAGQEEYSAMRDQYMRTGEGF
LCVFAINNTKSFEDIHHYREQIKRVKDSEDVPMVLVGNKCDLPSRTVDTKQAQDLARSYGIPFIETSAKTR
QGVDDAFYTLVREIRKHKEKMSKDGKKKKKKSATACVIM-

MBP-KRas A3A1 Forward: CGCCTGTGTCATAATGTGATAATAAG
MBP-KRas A3A1 Reverse: GTTGCGGATTTCTTTTTTTCTTCTTGC

GFP-HRAS WT

Plasmid: eGFP-C1

DNA Sequence:
ATGACGGAATATAAGCTGGTGGTGGTGGGCGCCGGCGGTGTGGGCAAGAGTGCGCTGACCATCCAGCTG
ATCCAGAACCATTTTGTGGACGAATACGACCCCACTATAGAGGATTCCTACCGGAAGCAGGTGGTCATT
GATGGGGAGACGTGCCTGTTGGACATCCTGGATACCGCCGGCCAGGAGGAGTACAGCGCCATGCGGGAC
CAGTACATGCGCACCGGGGAGGGCTTCCTGTGTGTGTTTGCCATCAACAACACCAAGTCTTTTGAGGAC
ATCCACCAGTACAGGGAGCAGATCAAACGGGTGAAGGACTCGGATGACGTGCCCATGGTGCTGGTGGGG
AACAAGTGTGACCTGGCTGCACGCACTGTGGAATCTCGGCAGGCTCAGGACCTCGCCCGAAGCTACGGC
ATCCCCTACATCGAGACCTCGGCCAAGACCCGGCAGGGAGTGGAGGATGCCTTCTACACGTTGGTGCGT
GAGATCCGGCAGCACAAGCTGCGGAAGCTGAACCCTCCTGATGAGAGTGGCCCCGGCTGCATGAGCTGC
AAGTGTGTGCTCTCCTGA

Protein sequence:
MTEYKLVVVGAGGVGKSALTIQLIQNHFVDEYDPTIEDSYRKQVVIDGETCLLDILDTAGQEEYSAMRDQ
YMRTGEGFLCVFAINNTKSFEDIHQYREQIKRVKDSDDVPMVLVGNKCDLAARTVESRQAQDLARSYGIP
YIETSAKTRQGVEDAFYTLVREIRQHKLRKLNPPDESGPGCMSCKCVLS

HRas GFP Forward: CGGGGTACCATGACGGAATATAAGCTGGTG
HRas GFP WT Reverse: CGCGGATCCTCAGGAGAGCACACACTTGCAGCTCATGCAG

GFP-HRAS G12D

Plasmid: eGFP-C1

DNA Sequence:
ATGACGGAATATAAGCTGGTGGTGGTGGGCGCCGGCGGTGTGGGCAAGAGTGCGCTGACCATCCAGCTG
ATCCAGAACCATTTTGTGGACGAATACGACCCCACTATAGAGGATTCCTACCGGAAGCAGGTGGTCATT
GATGGGGAGACGTGCCTGTTGGACATCCTGGATACCGCCGGCCAGGAGGAGTACAGCGCCATGCGGGAC
CAGTACATGCGCACCGGGGAGGGCTTCCTGTGTGTGTTTGCCATCAACAACACCAAGTCTTTTGAGGAC
ATCCACCAGTACAGGGAGCAGATCAAACGGGTGAAGGACTCGGATGACGTGCCCATGGTGCTGGTGGGG
AACAAGTGTGACCTGGCTGCACGCACTGTGGAATCTCGGCAGGCTCAGGACCTCGCCCGAAGCTACGGC
ATCCCCTACATCGAGACCTCGGCCAAGACCCGGCAGGGAGTGGAGGATGCCTTCTACACGTTGGTGCGT
GAGATCCGGCAGCACAAGCTGCGGAAGCTGAACCCTCCTGATGAGAGTGGCCCCGGCTGCATGAGCTGC
AAGTGTGTGCTCTCCTGA

Protein sequence:
MTEYKLVVVGADGVGKSALTIQLIQNHFVDEYDPTIEDSYRKQVVIDGETCLLDILDTAGQEEYSAMRDQ
YMRTGEGFLCVFAINNTKSFEDIHQYREQIKRVKDSDDVPMVLVGNKCDLAARTVESRQAQDLARSYGIP
YIETSAKTRQGVEDAFYTLVREIRQHKLRKLNPPDESGPGCMSCKCVLS

HRas GFP Forward: GGTGGGCGCCGACGGTGTGGGCA
HRas GFP WT Reverse: ACCACCAGCTTATATTCCGTCATTCAGGAGAGCACACAC

GFP-HRAS G12D I1

Plasmid: eGFP-C1

DNA Sequence:
ATGACGGAATATAAGCTGGTGGTGGTGGGCGCCGGCGGTGTGGGCAAGAGTGCGCTGACCATCCAGCTG
ATCCAGAACCATTTTGTGGACGAATACGACCCCACTATAGAGGATTCCTACCGGAAGCAGGTGGTCATT
GATGGGGAGACGTGCCTGTTGGACATCCTGGATACCGCCGGCCAGGAGGAGTACAGCGCCATGCGGGAC
CAGTACATGCGCACCGGGGAGGGCTTCCTGTGTGTGTTTGCCATCAACAACACCAAGTCTTTTGAGGAC

ATCCACCAGTACAGGGAGCAGATCAAACGGGTGAAGGACTCGGATGACGTGCCCATGGTGCTGGTGGGG
AACAAGTGTGACCTGGCTGCACGCACTGTGGAATCTCGGCAGGCTCAGGACCTCGCCCGAAGCTACGGC
ATCCCCTACATCGAGACCTCGGCCAAGACCCGGCAGGGAGTGGAGGATGCCTTCTACACGTTGGTGCGT
GAGATCCGGCAGCACAAGCTGCGGAAGCTGAACCCTCCTGATGAGAGTGGCCCCGGCTGCATGAGCTGC
ATCTGTGTGCTCTCCTGA

Protein sequence:
MTEYKLVVVGADGVGKSALTIQLIQNHFVDEYDPTIEDSYRKQVVIDGETCLLDILDTAGQEEYSAMRDQ
YMRTGEGFLCVFAINNTKSFEDIHQYREQIKRVKDSDDVPMVLVGNKCDLAARTVESRQAQDLARSYGIP
YIETSAKTRQGVEDAFYTLVREIRQHKLRKLNPPDESGPGCMSCICVLS

HRas GFP I1 Forward: GGTGGGCGCCGACGGTGTGGGCA
HRas GFP I1 Reverse: ACCACCAGCTTATATTCCGTCATTCAGGAGAGCACACAC

GFP-HRAS G12D A1

Plasmid: eGFP-C1

DNA Sequence:
ATGACGGAATATAAGCTGGTGGTGGTGGGCGCCGGCGGTGTGGGCAAGAGTGCGCTGACCATCCAGCTG
ATCCAGAACCATTTTGTGGACGAATACGACCCCACTATAGAGGATTCCTACCGGAAGCAGGTGGTCATT
GATGGGGAGACGTGCCTGTTGGACATCCTGGATACCGCCGGCCAGGAGGAGTACAGCGCCATGCGGGAC
CAGTACATGCGCACCGGGGAGGGCTTCCTGTGTGTGTTTGCCATCAACAACACCAAGTCTTTTGAGGAC
ATCCACCAGTACAGGGAGCAGATCAAACGGGTGAAGGACTCGGATGACGTGCCCATGGTGCTGGTGGGG
AACAAGTGTGACCTGGCTGCACGCACTGTGGAATCTCGGCAGGCTCAGGACCTCGCCCGAAGCTACGGC
ATCCCCTACATCGAGACCTCGGCCAAGACCCGGCAGGGAGTGGAGGATGCCTTCTACACGTTGGTGCGT
GAGATCCGGCAGCACAAGCTGCGGAAGCTGAACCCTCCTGATGAGAGTGGCCCCGGCTGCATGAGCTGC
GCCTGTGTGCTCTCCTGA

Protein sequence:
MTEYKLVVVGADGVGKSALTIQLIQNHFVDEYDPTIEDSYRKQVVIDGETCLLDILDTAGQEEYSAMRDQ
YMRTGEGFLCVFAINNTKSFEDIHQYREQIKRVKDSDDVPMVLVGNKCDLAARTVESRQAQDLARSYGIP
YIETSAKTRQGVEDAFYTLVREIRQHKLRKLNPPDESGPGCMSCACVLS

HRas GFP Forward: GGTGGGCGCCGACGGTGTGGGCA
HRas GFP WT Reverse: CGCGGATCCTCAGGAGAGCACACAGGCGCAGCTCATGCAGCC

GFP-HRAS G12D CS

Plasmid: eGFP-C1

DNA Sequence:
ATGACGGAATATAAGCTGGTGGTGGTGGGCGCCGGCGGTGTGGGCAAGAGTGCGCTGACCATCCAGCTG
ATCCAGAACCATTTTGTGGACGAATACGACCCCACTATAGAGGATTCCTACCGGAAGCAGGTGGTCATT
GATGGGGAGACGTGCCTGTTGGACATCCTGGATACCGCCGGCCAGGAGGAGTACAGCGCCATGCGGGAC
CAGTACATGCGCACCGGGGAGGGCTTCCTGTGTGTGTTTGCCATCAACAACACCAAGTCTTTTGAGGAC
ATCCACCAGTACAGGGAGCAGATCAAACGGGTGAAGGACTCGGATGACGTGCCCATGGTGCTGGTGGGG
AACAAGTGTGACCTGGCTGCACGCACTGTGGAATCTCGGCAGGCTCAGGACCTCGCCCGAAGCTACGGC
ATCCCCTACATCGAGACCTCGGCCAAGACCCGGCAGGGAGTGGAGGATGCCTTCTACACGTTGGTGCGT
GAGATCCGGCAGCACAAGCTGCGGAAGCTGAACCCTCCTGATGAGAGTGGCCCCGGCTGCATGAGCTGC
AAGTCTGTGCTCTCCTGA

Protein sequence:
MTEYKLVVVGADGVGKSALTIQLIQNHFVDEYDPTIEDSYRKQVVIDGETCLLDILDTAGQEEYSAMRDQ
YMRTGEGFLCVFAINNTKSFEDIHQYREQIKRVKDSDDVPMVLVGNKCDLAARTVESRQAQDLARSYGIP
YIETSAKTRQGVEDAFYTLVREIRQHKLRKLNPPDESGPGCMSCKSVLS

HRas GFP CS Forward: GGTGGGCGCCGACGGTGTGGGCA
HRas GFP CS Reverse: CGCGGATCCTCAGGAGAGCACAGACTTGCAGCTCATGCAGC

References

1. Pylypenko O, Rak A, Durek T, et al. Structure of doubly prenylated Ypt1:GDI complex and the mechanism of GDI-mediated Rab recycling. *EMBO J*. 2006;25(1):13-23. doi:10.1038/sj.emboj.7600921
